# G9a an Epigenetic Therapeutic Strategy for Neurodegenerative Conditions: From Target Discovery to Clinical Trials

**DOI:** 10.1002/med.22096

**Published:** 2025-01-06

**Authors:** Aina Bellver‐Sanchis, Marta Ribalta‐Vilella, Alba Irisarri, Pinky Gehlot, Bhanwar Singh Choudhary, Abhisek Jana, Vivek Kumar Vyas, Deb Ranjan Banerjee, Mercè Pallàs, Ana Guerrero, Christian Griñán‐Ferré

**Affiliations:** ^1^ Department of Pharmacology and Therapeutic Chemistry Institut de Neurociències‐Universitat de Barcelona Barcelona Spain; ^2^ Department of Pharmaceutical Chemistry Institute of Pharmacy Nirma University Ahmedabad India; ^3^ Department of Pharmacy Central University of Rajasthan Ajmer India; ^4^ Drug Discovery and Development Centre (H3D) University of Cape Town Rondebosch South Africa; ^5^ Department of Chemistry National Institute of Technology Durgapur Durgapur India; ^6^ Instituto de Salud Carlos III, Centro de Investigación en Red, Enfermedades Neurodegenerativas (CIBERNED) Madrid Spain

**Keywords:** aging, clinical trials, EHMT2, epigenetics, G9a, neurodegenerative conditions, small‐molecules

## Abstract

This review provides a comprehensive overview of the role of G9a/EHMT2, focusing on its structure and exploring the impact of its pharmacological and/or gene inhibition in various neurological diseases. In addition, we delve into the advancements in the design and synthesis of G9a/EHMT2 inhibitors, which hold promise not only as a treatment for neurodegeneration diseases but also for other conditions, such as cancer and malaria. Besides, we presented the discovery of dual therapeutic approaches based on G9a inhibition and different epigenetic enzymes like histone deacetylases, DNA methyltransferases, and other lysine methyltransferases. Hence, findings offer valuable insights into developing novel and promising therapeutic strategies targeting G9a/EHMT2 for managing these neurological conditions.

## 1 Introduction

Neurodegenerative diseases represent signifsuicant causes of disability and mortality globally [1]. Despite extensive research spanning decades, most strategies to reverse these conditions merely address symptoms and fail to impede disease progression. The recent advancements in sequencing technologies have facilitated the mapping of transcriptomic patterns in a multitude of neurodegenerative disorders, shedding light on classical neurodegenerative pathways and revealing novel targets, including synaptic degeneration, a common characteristic in many of these diseases [2, 3]. Moreover, despite comprehensive genetic analyses involving large patient cohorts, a considerable proportion of neurodegenerative cases remain genetically unresolved. [4]. Consequently, several studies on neurodegenerative diseases emphasize the impact of adverse environmental factors, which are recognized as contributors to neurodegeneration and the subsequent development of pathological and behavioral abnormalities [5, 6, 7].

**Figure 1:**
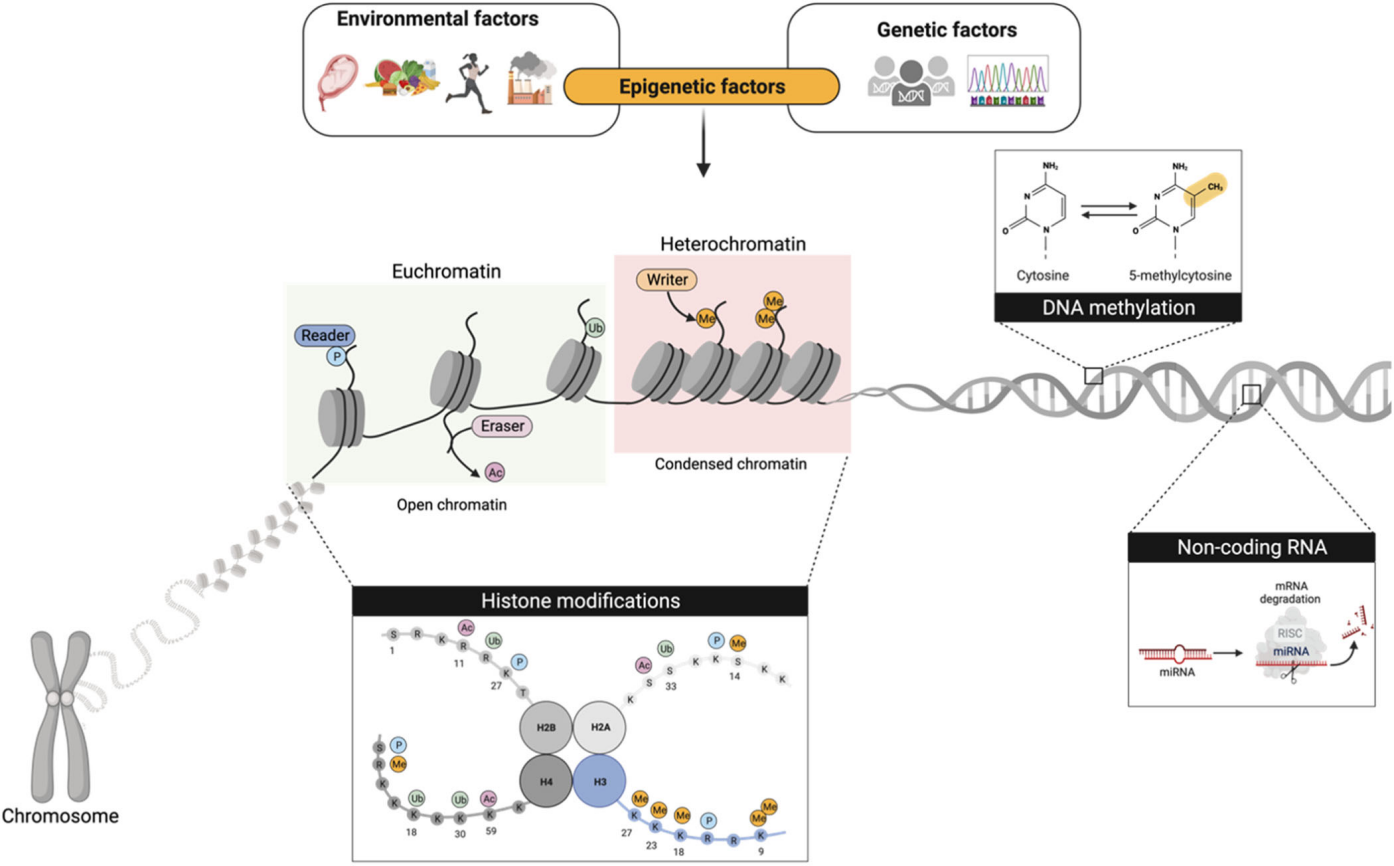
Schematic representation of epigenetics and its key mechanisms linking the genome to environmental influences. The diagram illustrates three main epigenetic processes: DNA methylation, histone modifications, and noncoding RNA regulation. [Color figure can be viewed at wileyonlinelibrary.com]

In this context, epigenetics is pivotal as a link between the genome and the environment (Figure 1). It is defined as a branch of genetics focused on studying heritable and reversible changes in gene expression without altering the DNA sequence [8]. Over the past few decades, several epigenetic mechanisms have been thoroughly studied, making them key targets for drug development, particularly in the field of neuroscience. Aberrant epigenetic marks can silence genes crucial for neuronal function while upregulating genes associated with disease progression [9, 10, 11]. Thus, epigenetic therapy is based on the idea that manipulating epigenetic changes can restore normal gene function, potentially slowing down or halting the progression of neurodegenerative diseases. Currently, numerous epigenetic targets are under investigation for treating neurodegenerative diseases [12, 13, 14]. Our research group is deeply intrigued by the histone methyltransferase called G9a, which seems to play a significant role in the context of neurodegeneration. In the present review, we delve into the findings regarding G9a, exploring the impact of its pharmacological and/or gene inhibition in various neurodegenerative disorders, including Alzheimer's disease (AD), Parkinson's disease (PD), Huntington's disease (HD), autism spectrum disorder (ASD), and neuropsychiatric disorders [15, 16, 17]. Additionally, we examine the advancements in the design and synthesis of G9a inhibitors, which hold promise not only as treatments for neurodegenerative diseases but also for other conditions such as cancer [18, 19].

## 2 Epigenetic Mechanisms

At present, different epigenetic changes have been identified, such as DNA methylation (5‐mC) or posttranslational modifications (PTMs) of nucleosomal histones (Figure 1). These modifications alter chromatin structure or recruit histone modifier enzymes, leading to changes in gene expression. Walther Flemming initially introduced the concept of chromatin, and it is composed of nucleosomes, each of them consisting of an octamer of histones (H) (H2A, H2B, H3, and H4) wrapped around 147 base pairs of DNA and the linker histone H1 [20, 21, 22]. The location and configuration of nucleosomes impact the functionality of chromatin. Heterochromatin refers to densely packed chromatin, which restricts the accessibility of proteins and transcriptional machinery to the gene promoter regions. However, when chromatin adopts a relaxed state called euchromatin, modifications and transcription processes are allowed [22]. Hence, this highly dynamic 3D architecture orchestrates the intricate organization of the genome, acting as an epigenetic regulator of gene expression. Furthermore, microRNAs (miRNAs) are a class of noncoding RNAs (ncRNAs) that exert regulatory control over their mRNA targets via degradation and/or translational repression and consequently control gene expression posttranscriptionally [23] (Figure 1).

### 2.1 DNA Methylation and Hydroxymethylation

Among the epigenetic changes, DNA methylation of the 5‐carbon of a cytosine residue (5‐mC) in cytosine‐guanine dimers (CpGs) is the only one that directly modifies the DNA [24, 25]. These CpG island repeats are very close to gene regulatory regions, and they commonly appear in the promoters (mostly at housekeeping and transcription start sites). Thus, the effect of 5‐mC is to silence gene expression, interfering with transcriptional initiation. This methylation process is catalyzed by the family of methyltransferases, which are collectively referred to as DNA methyltransferases (DNMTs). Three distinct types of DNMTs have been identified in mammals: DNMT1, DNMT3a, and DNMT3b. Although the different DNMTs share a similar mechanism, they have different roles. DNMTs are either maintenance (DNMT1) or de novo (DNMT3A and DNMT3B) methyltransferases [24, 26]. MBD1, MBD2, MBD3, and MeCP2 are methyl‐binding proteins that recognize 5‐mC and recruit histone modifiers, so the chromatin remodeling process is enabled [20, 21]. Furthermore, hydroxymethylation of cytosines (5‐hmC) is another modification with a direct impact on the DNA, potentially as an intermediary step preceding the demethylation of cytosine. Enzymes called “Ten‐Eleven Translocation family” (TETs), notably TET1, TET2, and TET3, are responsible for this modification [27].

### 2.2 Histone Modifications

Histones are pivotal proteins in transcription processes that are susceptible to PTMs, including acetylation, methylation, phosphorylation, ubiquitination, sumoylation, ADP‐ribosylation, citrullination, glycosylation, hydroxylation, and isomerization [28].

Among these, the two main modifications are acetylations and methylations. On the one hand, chromatin structure is remodeled by histone acetylation, which was one of the earliest histone modifications to be described. Histone acetyltransferases (HATs) constitute the group of enzymes responsible for adding acetyl‐CoA to the lysine (K) residues of H2A, H2B, H3, and H4 histone proteins [29, 30]. The addition of acetyl neutralizes the positive charge of lysine amino acids, making DNA and histones repel each other. This way, nucleosomes turn into a relaxed state in which transcription is activated. In contrast, histone deacetylases (HDACs), also known as lysine deacetylases (KDACs), erase the acetylation mark regulating transcription negatively and promoting gene silencing. There are three main classes of HATs: the GANT family, the CBP/p300 family, and the MYST family. Additionally, four classes of HDACs (I, Iia, Iib, III, and IV) have been described. Balance between both enzyme groups is needed for controlling gene expression and, especially, DNA repair; if the DNA is damaged and acetylation is dysregulated, it can lead to cell death [30, 31].

On the other hand, histone methylation accounts for the vast majority of the epigenetic marks that control gene expression through activating or repressing specific genes [32]. Histone methylation occurs at lysine (K) or arginine I residues, which act either as transcriptional activators or repressors. This modification is more stable than acetylation. Both K and R residues undergo different methylations depending on the degree of modification presented. K can be mono‐ (me1), di‐ (me2), and tri‐methylated (me3), while R is only methylated with one or two methyl groups both symmetrically and asymmetrically (me1 and me2s or me2a, respectively) [32, 33], However, histidine has also been reported to be monomethylated, although not much research has focused on its repercussions on gene expression regulation. These PTMs are chemically catalyzed by numerous enzymes classified according to their target. Histone methyltransferases (HMTs), also known as writers, are responsible for adding the methyl group to histone protein residues. For the purposes of this review, histone lysine methyltransferases (KMTs) are of interest as they transfer a methyl group from the donor S‐adenosyl‐l‐methionine (SAM) to a lysine residue on histones, specifically to the ε‐nitrogen atom [34, 35]. KMTs are divided into two groups depending on their catalytic methyltransferase domain sequence, known as SET‐containing and non‐SET domains. The first group, conserving the SET sequence, includes SUV39H1/2, G9a, GLP, SMYD, SETDB1, and EZH2, among others [30]. H3 and H4 proteins, the main targets of these enzymes, present the most relevant methylation marks involved in gene transcription regulation. In particular, K methylation of both histones (H3K4, H3K9, H3K27, H3K36, H3K79, and H4K20) modulates gene expression, either activating or silencing its transcription, depending on the degree of modification [35]. Moreover, arginiI(R) methyltransferases (PRMTs), which target R residues on histones, are also divided into three main groups. The first type, including PRMT1, PRMT2, PRMT3, PRMT4, PRMT6, and PRMT8, catalyzes me2a. The second one, including PRMT5 and PRMT9, incorporates me2s [30]. Lastly, PRMT7 monomethylates R residues [35]. Arginine methylations of H3 and H4 (H3R2, H3R8, H3R17, H3R26, and H4R3) are involved in transcription regulation [36] (detailed in Table 1). As a result, methylation offers the greatest variability of epigenetic changes at the gene expression level, from chromatin structure to on‐target loci through the interaction of cell‐specific transcription, initiation, and elongation factors.

**Table 1:** Histone modifications associated with various neurological disorders.

Neurodegenerative diseases	Chromatin remodeling modification	Writers	Erasers	Reference
Alzheimer's disease (AD)	H4 acetylation ↓	HATs	HDACs	[37, 38, 39]
	H3K27ac ↑	HATs	HDACs	[40, 41, 42, 43]
	H3K9ac ↑	HATs	HDACs	[41, 42, 43]
	—	—	HDAC1 ↓	[44]
	—	—	HDAC2 ↓	[45]
	—	—	HDAC3↓	[46, 47]
	—	—	HDAC6 ↓	[48]
	H3K9me2 ↑	G9a/GLP	KDMs	[41, 42, 43]
	H3K27me3 ↑	G9a/GLP	KDMs	[41, 42, 43]
	H3K4me2/3 ↑	SETD1A/B	KDMs	[41, 42, 43]
	DNA methylation ↑	DNMT1, 3a, 3b	TETs	[41, 42, 43]
Parkinson's disease (PD)	H3K14ac ↑	HATs	HDACs	[49]
	H3K18ac ↑	HATs	HDACs	[49]
	H3K9me1/2 ↑	G9a/GLP	KDMs	[50]
	—	—	HDAC2 ↑	
	DNA methylation	—	TET2 ↑	[50]
	H3K27ac ↑	HATs	HDACs	[50, 51]
	H3K4me3 ↑	SETD1A/B	KDMs	[50, 51]
Huntington's disease (HD)	H3K9me2,3 ↑	G9a/GLP	KDMs	[52]
	H3K4me3 ↓	SETD1A/B	KDMs	[53]
	—	—	HDAC1 ↑	[54]
	—	—	HDAC2 ↑	[55]
	—	—	HDAC3 ↑	[54, 56]
	—	—	HDAC4 ↑	[57]
	H2AFY ↑	—	—	[58]
Autism spectrum disorder (ASD)	H3K9me2,3 ↑	G9a/GLP	KDMs	[59, 60]
	H3K4me3 ↓	SETD1A/B	KDMs	[59, 60, 61]
Schizophrenia (SZ)	H3K9me2 ↑	G9a/GLP	KDMs	[59]
	DNA methylation ↑	DNMT1, 3a, 3b	TETs	[62]
	—	—	HDAC1 ↑	[59, 62]
	—	—	HDAC2 ↓	[62]
	H3K4me3 ↓	SETD1A/B	KDMs	[59]
	H3meR17 ↑	—	—	[59]
	H3K9ac↓	HATs	HDACs	[59]
	H3K14ac ↓	HATs	HDACs	[59]
Major depressive disorder (MDD)	H3K9me2 ↑	G9a/GLP	KDMs	[63]
	DNA methylation ↑	DNMT1, 3a, 3b	TETs	[63]
	H3K14ac ↑	HATs	HDACs	[63]
	—	—	HDAC2 ↓	[63]
	H3K4ac ↑	HATs	HDACs	[63]
	H3K4me3 ↑	SETD1A/B	KDMs	[63]
	H3K27me3 ↑	G9a/GLP	KDMs	[63]
	H3K9ac ↓	HATs	HDACs	[59]

*Note:* This comprehensive table summarizes key epigenetic changes observed in different neurological conditions.

Moreover, methylation not only affects histones forming chromatin structures but also free histones and non‐histone proteins [64]. In contrast, histone demethylases (HDMs) or erasers, reported afterward, demonstrated that methylation is a dynamic reversible process, and its biological outcome depends on the replacement or establishment of the methylation [64, 65]. The presence or absence of the mark is recognized by reader proteins activating downstream signaling for gene activation or repression and, therefore, the combination of distinct methylation marks provides a wide variety of epigenetic regulation mechanisms, involving cell cycle regulation, DNA damage repair, stress response, and cell differentiation during brain development [64].

Last but not least, additional modifications take part in relevant biological processes including phosphorylation, ubiquitylation, SUMOylation, glycosylation, or ADP‐ribosylation [66, 67]. All of them play a key role in maintaining genome stability by preventing chromosome dysregulation [33, 68], which highlights the importance of histone modifications concerning disease. The latest developments in mass spectrometry have recently provided insights into new histone modifications, including crotonylation, lactylation, and serotonylation. These modifications have been shown to be disrupted in neurodevelopment [69].

### 2.3 Noncoding RNA Regulation

ncRNAs are a class of RNA molecules that are transcribed from DNA but do not undergo translation into functional proteins, and they are clustered depending on their cellular function [70]. A limited proportion of the genome, representing only 1.5%, is transcribed to mRNA. The remainder is primarily regarded as functional ncRNAs, which are categorized into two groups based on size, designated as either long or short. Among these, the most intriguing are microRNAs (miRNAs), which are 18–25 nucleotides in length and are associated with gene repression, or the blocking of transcription. Indeed, miRNAs function via RNA interference (RNAi), resulting in the degradation of the target mRNA or inhibition of translation. [71]. Furthermore, miRNAs regulate the development, maturation, differentiation and apoptosis of the cell, cell signaling, cellular interactions, and homeostasis [72, 73]. As a consequence, the dysregulation of miRNA–epigenetic mechanisms contributes to a variety of diseases, such as cancer [74, 75], cardiovascular disease [76, 77], diabetes [78, 79], neurodegenerative diseases [80, 81], and age‐related cognitive decline [82, 83]). Of note, there are other ncRNA regulators such as piwi‐interacting RNAs (piRNAs) and long noncoding RNAs (lncRNAs).

## 3 Chromatin Remodeling in Neurodevelopment

A better understanding of chromatin remodeling mechanisms will foster the development of new drug targets for improved pharmaceutical interventions. The mechanisms that drive chromatin remodeling can be classified into two main categories: (1) covalent modifications of chromatin components and (2) noncovalent modifications involving ATP‐dependent chromatin remodeling complexes (CRCs) [84]. Covalent modifications mainly involve PTMs and DNA alterations, as previously discussed [21, 85]. In fact, one of the main functions of these remodelers is to relax the chromatin structure, allowing both DNA and histones to become accessible to transcription factors and posttranslational modifiers. Chromatin remodeling facilitates changes in gene expression linked to synaptic plasticity during both development and adulthood [86]. This process enables the collaboration of genetic predisposition, experiences, and neuronal activity, modifying cellular and behavioral responses. Moreover, recent discoveries highlight epigenetic dysregulation, particularly histone lysine methylation, in cognitive deficits and neuronal death as part of neurodegenerative diseases [34, 87]. Among all the chromatin remodelers, in this review, we focus on the effects of G9a‐mediated methylation and its role in neurological diseases.

## 4 Chromating Reprogramming

Chromatin reprogramming plays a crucial role in the pathogenesis and progression of neurodegenerative diseases, offering new insights into these complex disorders and potential therapeutic avenues. Epigenetic mechanisms, including DNA methylation, histone modifications, and chromatin remodeling, have been demonstrated to play a role in a number of key aspects of neuronal function and development. Recent discoveries have highlighted the critical functions of chromatin in the aging brain. This has led to an emerging realization that the maintenance of healthy brain function relies heavily on epigenetic mechanisms [84, 86, 87]. In neurodegenerative conditions such as AD, PD, HD, and neuropsychiatric disorders, aberrant chromatin remodeling and histone modifications disrupt normal transcriptional regulation, contributing to neuronal dysfunction [23]. For instance, altered histone methylation patterns are observed in AD brains; also, dysregulation of G9a is linked to several neurodegenerative diseases. As aforementioned, specific chromatin regulators have emerged as key players in the neurodegenerative process. These include REST (RE1‐Silencing Transcription factor), polycomb repressive complex 2 (PRC2), and histone deacetylases (HDACs). Their dysregulation can lead to widespread changes in gene expression profiles, affecting neuronal survival and function.

Additionally, age‐related changes in chromatin states, such as increased H4K16 acetylation, may contribute to the risk of developing neurodegenerative diseases [41]. Recent studies have shown that both activating and repressing histone methylation marks, particularly methylation of H3K4, H3K9, and H3K27 residues, are affected in neurodegenerative disorders and might contribute to transcriptional abnormalities [35, 40, 53, 61, 88, 89, 90, 91]. Importantly, pharmacological inhibition of certain methyltransferase or demethylase enzymes has shown promise in improving pathology in disease models. In neurodegenerative diseases, particularly AD, G9a has been found to play a significant role in disease progression. In fact, G9a activity leads to aberrant histone methylation patterns, which in turn contribute to transcriptional dysregulation of genes involved in synaptic plasticity, neuronal function, and survival [92, 93, 94, 95, 96]. These topics will be discussed in more detail below.

## 5 G9a, a Lysine Methyltransferase

Protein KMTs (PKMTs) catalyze the transfer of a methyl group from the SAM donor to the ε‐nitrogen of a K residue on protein substrates. Notably, the ε‐amino group of Ks can accept up to three methyl groups, leading to the formation of mono‐ (me), di‐ (me2), or tri‐methylation (me3) [97, 98]. To date, two PKMT families have been identified: the SET lysine methyltransferases, which constitute the majority of PKMTs [97], and the seven β‐strand methyltransferase (7βS) or class I family [99].

### 5.1 Discovery of G9a

G9a, identified and sequenced in 1990, is a PKMT that catalyzes methyl group transfer to K residues via the SET domain, and is likely the main enzyme responsible for the mono‐ and di‐methylation of H3 lysine 9 (H3K9me1 and H3K9me2, respectively) in euchromatin. G9a is also known as lysine methyltransferase‐1C (KMT1C), euchromatic histone *N*‐methyltransferase 2 (EHMT2), or BAT8 (HLA‐B–associated transcript 8) [34].

The SET domain was originally identified in *Drosophila melanogaster* [100] and was named after the recognition of a 130 amino acid sequence at the C‐terminal end of three gene regulatory factors: Su(var)3‐9, Enhancer of zeste, and Triothorax [101]. The first discovery of a SET domain–containing histone lysine methyltransferase was Suv39h1, thus elucidating the association of the SET domain with lysine methylation activity [102]. Shortly after, G9a emerged as the second protein within the group containing the SET domain of PKMT [98]. A paralog of G9a has been identified and is known as G9a‐like protein (GLP), lysine methyltransferase‐1D (KMT1D), or euchromatic histone *N*‐methyltransferase 1 (EHMT1). Approximately 45% of the amino acid sequence of G9a is identical to that of GLP, with a sequence similarity of ~70% [103]. Their primary difference lies in the N‐terminus as opposed to the SET domain, where they maintain a high level of conservation sharing with over 80% sequence identity [97].

### 5.2 G9a Structure

G9a mRNA has two isoforms (isoform A, long isoform; isoform B, short isoform) resulting from alternative splicing of its exon 10 [104]. Isoform A is the full‐length G9a protein encompassing 1210 amino acids and is a product of two transcripts: the G9a gene (comprising 24 exons) and the NG36 gene (containing 4 exons). Isoform B, which represents a splice variant resulting from the excision of exon 10, consists of 1176 amino acids [98]. Alternative splicing was identified across all tissues although the ratio between both isoforms varies. Specifically, G9a isoform B predominates in the kidney, thymus, and testis. A significant accumulation of isoform B was also observed in epithelial cell lines compared to both highly transformed cell lines and mesenchymal cell lines. Although no catalytic changes were reported following exon 10 inclusion, alternative splicing might influence subcellular localizations [105]. The G9a mouse homolog, located on chromosome 17, also presents two alternatively spliced isoforms. The long isoform (G9a L) lacks exon 1 but retains exon 2, while the short isoform (G9a S) contains exon 1 but excises a portion of exon 2 [103].

As aforementioned, G9a is a member of the Su(var)3‐9 family of methyltransferases, and a defining feature of this family is the highly conserved SET domain at the C‐terminal region [106, 107]. In fact, this SET domain accounts for the methyltransferase function and enables protein–protein interactions thanks to its ankyrin repeats [108, 109]. The human G9a protein exhibits other well‐defined domains, including a predominantly disordered N‐terminal region, and a cysteine‐rich region (CRR) [110].

The SET domain consists of a sequence of β strands that fold into three sheets, encircling a knot‐like structure [111]. The conserved nucleus of the SET domain is flanked by a pre‐SET (nSET) and a post‐SET (cSET) domain. The pre‐SET domain reinforces structural stability through interactions with various structures of the core SET domain. Additionally, a post‐SET domain is responsible for establishing a hydrophobic channel through the involvement of an aromatic residue. In contrast to the SET domain, the pre‐SET and post‐SET domains are not conserved sequences [112]. G9a adopts a typical fold, comprising a conserved SET domain and a variable i‐SET insert. The i‐SET domain functions as a docking platform for the H3 tail, forming a conserved pair of hydrogen bonds between the i‐SET domain and the K residue, as well as crucial contacts with a basic side chain located upstream of the methyl acceptor. This is likely to ensure the correct positioning of the peptide as lysine enters the active site [110].

The pre‐SET domain comprises nine conserved cysteine residues, organized into two groups, one with five cysteines and the other with four cysteines, separated by varying numbers of amino acids. These nine cysteines coordinate the formation of an equilateral triangular cluster with three zinc ions. Additionally, a fourth zinc ion is located proximate to the SAM binding site. These four structural zinc fingers play a pivotal role in ensuring proper protein folding and enzymatic activity. In the post‐SET region, three conserved cysteine residues are noted, and they appear to be crucial for KMT activity within the SUV39 family [107].

The ankyrin repeat domain acts as a binding module for mono‐ and di‐methylated lysine, serving as a reader domain critical for protein–protein interactions. Ankyrin repeats adopt helix‐turn‐helix‐β‐turn structures, with the H3 peptide interposed between β‐turns and helices within the fourth and fifth ankyrin repeats [113]. G9a ankyrin repeats exhibit specific binding to K9‐methylated peptides, displaying no affinity for either K4‐ or K27‐methylated peptides. Interestingly, GLP and its ankyrin repeats show similar binding patterns to various modified H3 peptides as G9a, with the distinction that GLP displays stronger affinity for H3K9me1 than for H3K9me2 [113].

Finally, the CRR domain encompasses an array of conserved cysteine and histidine residues. It assumes a compact fold, and it is associated with four bound zinc atoms. The C‐terminal of the CRR keeps an interesting new gene (RING) domain fold. Typically, RING domains function as protein ubiquitin ligases [110]. Based on existing experimental evidence, it is likely that CRR operates as a novel protein‐binding domain facilitating interactions with several other proteins. Notably, these interactions involve cyclin D1, NRSF/REST, the Mediator complex, and ZNF200, ultimately recruiting EHMT proteins to specific chromatin loci [110, 114, 115, 116, 117].

### 5.3 G9a Activity

As mentioned above, SET‐domain proteins exhibit distinctive characteristics compared to other SAM‐dependent methyltransferases. Notably, the binding sites for the histone substrate and SAM are located on opposite sides of the SET domain. These substrate‐binding clefts are connected by a deep channel that traverses the core of the SET domain, facilitating the transfer of the methyl group from SAM to the ε‐amino group of the lysine substrate. This unique arrangement of substrate binding sites was originally proposed to allow multiple rounds of lysine methylation without dissociation of the protein substrate from the SET domain [118]. Within the SET domain, the tyrosine residue Y1154 plays an essential role in the catalytic activity of G9a. This tyrosine likely facilitates the deprotonation of the positively charged ammonium group, thereby favoring the methylation process [119].

Biochemical characterization of G9a and GLP has revealed that they form both homomeric and heteromeric complexes through their SET domains. However, in various human and mouse cells, endogenous molecules predominantly exist as the stoichiometric G9a–GLP heteromeric complex [120]. Complementation experiments showed that the enzymatic activity of G9a plays a more crucial role in in vivo KMT function than that of GLP. While it remains unclear why G9a and GLP cannot function in isolation as a KMT in vivo, there are several potential mechanistic explanations for the prevalence of the G9a–GLP heterocomplex within cells, despite their ability to form also homodimers [91]. Beyond its enzymatic activity, G9a serves as a molecular scaffold, forming complexes with other epigenetic regulators such as DNMTs and histone modifiers like EZH2. These interactions facilitate coordinated gene silencing and demonstrate the crosstalk between different epigenetic modifications.

### 5.4 G9a Function

In 2001, Tachibana et al. [98] identified histone proteins as the first substrates of G9a. They showed that G9a adds methyl groups to K9 and K27 on H3, unlike Suv39h1, which exclusively methylates K9 [89, 121]. Moreover, compared to Suv39h1, G9a has higher efficiency in transferring methyl groups to H3 in vitro. G9a has been recognized as the major PKMT for catalyzing mono‐ and di‐methylation of H3K9 [108], well‐known for its role in transcriptional silencing [91, 122, 123]. Thus, G9a acts as a writer of repressive histone marks but also interacts extensively with epigenetic readers and other regulatory complexes to coordinate gene silencing and chromatin organization. For instance, new evidence also points to the repressive role of H3K27 methylation by G9a [88, 89]. G9a also methylates H3 at K56 to ensure proper DNA replication, a methylation event induced by DNA damage. Furthermore, Vinson et al. [124] in 2022 reported that G9a can catalyze the addition of mono‐, di‐, and tri‐methylation on H3K18 and mono‐ and demethylation on H3K23. Similarly, G9a exhibits variant‐specific methylation of histone H1. H1.4 undergoes mono‐ and di‐methylation at H1.4K26, creating a binding site for HP1 and indicating a repressive role in transcription [125]. Conversely, G9a demethylates H1.2 at K187 and, intriguingly, this modification is not recognized by HP1 proteins [126]. Notably, Weiss et al. [126] demonstrated that G9a does not directly bind to methylated histone variants, suggesting a mechanism distinct from that observed in H3K9me1/2 methylation.

Another essential function of G9a is interacting with one of the most critical readers, the bromodomain‐containing protein 4 (BRD4). G9a and BRD4 exhibit a complex interplay in regulating gene expression and cellular differentiation, particularly in skeletal myogenesis. These epigenetic regulators have opposing roles: BRD4 promotes myogenic differentiation genes, while G9a represses them. Their interaction is characterized by mutual regulation, where the absence of one factor affects the activity of the other [127, 128]. For instance, BRD4 knockdown increases G9a activity, impairing myogenic differentiation. Conversely, inhibiting G9a can rescue differentiation defects caused by BRD4 absence [127]. The balance between these factors is crucial for precise transcriptional control. BRD4 inhibition increases G9a protein levels and occupancy at specific genomic loci, along with H3K9me2 marks. In contrast, G9a inhibition enhances BRD4 occupancy and H3K9ac marks at myogenic promoters [127, 128]. This delicate equilibrium between BRD4, an acetylation mark reader, and G9a, a methylation mark writer, fine‐tunes gene expression during cellular differentiation processes. This intricate regulatory relationship underscores the complexity of epigenetic control in development and potentially in disease contexts where these factors are implicated. Understanding the G9a–BRD4 interaction provides insights into the nuanced mechanisms of chromatin reprogramming and gene regulation, which could have implications for developmental biology and targeted therapies in pathological conditions [127, 128, 129].

A growing number of non‐histone proteins involved in various biological functions have been identified as G9a substrates [103]. Although the functional significance of methylation in non‐histone proteins remains unclear, G9a‐mediated methylation negatively influences the transcriptional activity of several transcription factors and proteins, including C/EBPb [130], MyoD [131], MEF2D [132], p53 [133], and GMFB [134]. Additionally, certain mediators involved in hypoxia response, such as Pontin, Reptin, and HIFα, are also subject to regulation through G9a‐mediated methylation [135, 136, 137]. Notably, G9a methylation activity affects other methyltransferases, like mAM, suggesting complex regulatory networks [103].

## 6 Role of the G9a in Neurodegenerative Diseases

Notably, G9a has become a promising target in the realm of neurodegenerative disease treatment. Its pivotal role in gene expression regulation and chromatin structure places it as a significant contributor to various cellular functions, including those critical for synaptic plasticity, neuronal function, and survival. Therefore, modulating its activity may have the potential to alter the disease progression. Here, we delve into G9a's role and explore the outcomes achieved by its modulation using both pharmacological and gene editing approaches in different neurodegenerative and neuropsychiatric conditions.

### 6.1 Alzheimer's Disease

AD is the most common neurodegenerative disease worldwide, and the incidence is expected to increase to over 91 million people worldwide by 2050 [138]. AD is often categorized into two types: early‐onset AD (EOAD) and late‐onset AD (LOAD). EOAD accounts for about 5%–10% of all AD cases that develop cognitive impairment before age 65; the remaining 90% of cases are diagnosed as LOAD and develop cognitive impairment after age 65 [139]. Around 1% of AD cases are single‐gene disorders, characterized by early onset and an autosomal dominant pattern of inheritance. Unfortunately, most cases of AD present a complex etiology as the exact mechanism and molecular basis of AD remain unclear. However, emerging evidence suggests a crucial role of environmental and genetic factors, as well as their complex interactions, in the development of AD [140].

Patients with AD manifest progressive loss of episodic memory and cognitive function, leading to subsequent deficits in language and visual skills, often accompanied by behavioral disturbances [141]. At the molecular level, the pathogenesis is characterized by two specific hallmarks: the β‐amyloid (Aβ) plaque deposition and neurofibrillary tangles (NFTs). On one hand, Aβ peptide is the product of amyloid precursor protein (APP) cleavage by β‐secretase (BACE1) and γ‐secretase (presenilin, nicastrin, anterior pharynx‐defective 1, and presenilin enhancer‐2). Its overproduction drives Aβ to self‐assemble into oligomers and, in turn, form toxic Aβ plaques [142]. On the other hand, NFTs are associated with an abnormally hyperphosphorylated and aggregated form of tau. Microtubule‐associated protein tau (MAPT) promotes microtubule assembly and stability, as well as membrane transport. Since cellular clearance systems begin to fail with age, Aβ and tau proteins accumulate in the brains of those with AD, causing neurodegeneration, synaptic lesions, and defects in neurogenesis, resulting in cognitive deficits [143].

Interestingly, there is evidence that the abnormal expression of genes related to memory and synaptic plasticity in AD could be due to an imbalance in epigenetic mechanisms [144, 145, 146]. These findings highlight the therapeutic potential of alternative mechanisms and pathways, such as epigenetic modifications, to treat AD. As we pointed out before, aberrant histone methylation is widely associated with AD pathogenesis. Moreover, postmortem AD brains show large‐scale changes in histone modifications. Importantly, G9a is upregulated in the AD brain, which correlates with both an increase in the repressive mark H3K9me2 and the Aβ concentration [134, 147]. Thus, if increased activity of G9a would play a causal role in AD, then, G9a inhibitors might be a suitable therapeutic approach.

**Figure 2:**
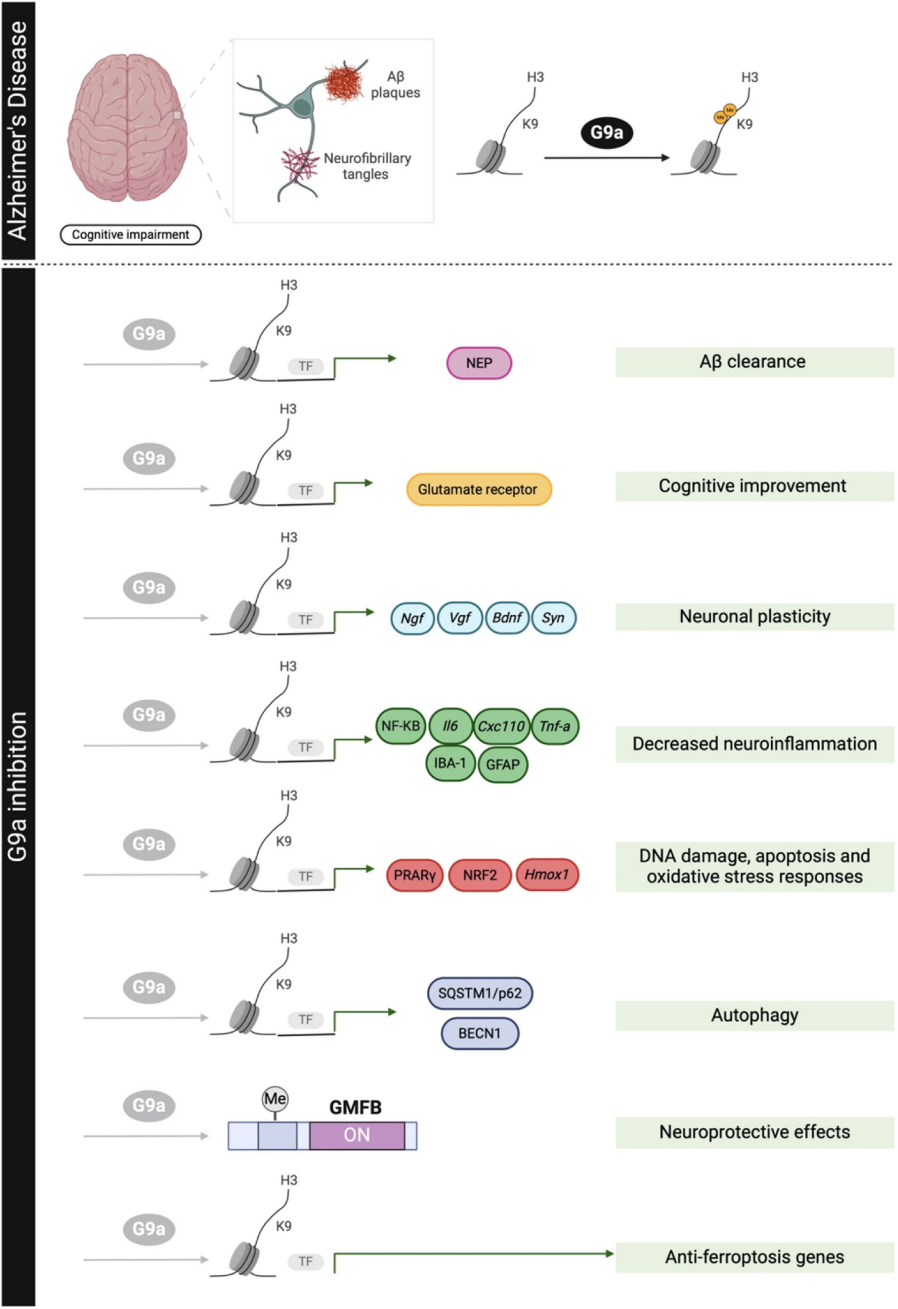
Comprehensive overview of findings in Alzheimer's disease (AD) following pharmacological inhibition or genetic modification of G9a. This schematic diagram illustrates the multifaceted effects of G9a modulation on various pathological aspects of AD. [Color figure can be viewed at wileyonlinelibrary.com]

Early studies in vitro showed that primary cortical and hippocampal neurons transfected with G9a siRNA in C57BL6 mice promoted hypoxia‐induced downregulation of G9a upregulation and H3K9me2 expression, restoring levels of neprilysin (NEP), an enzyme vital for Aβ clearance [148]. Moreover, inhibiting G9a/GLP with BIX‐01294 restored excitatory postsynaptic currents in Aβ‐treated human stem cell–derived neurons expressing high levels of G9a [149]. Additionally, pharmacological inhibition of G9a/GLP (BIX‐01294) decreases Aβ‐induced deficits in long‐term plasticity and synaptic tagging/capture in hippocampal pyramidal neurons [96], suggesting the G9a/GLP complex as a potential target to prevent Aβ‐induced plasticity deficits in hippocampal neurons (Figure 2).

Moreover, elevated G9a and GLP in the prefrontal cortex and hippocampus in an aged AD mouse model correlate with reduced glutamate receptor transcription and AD‐like cognitive deficits [147]. Noteworthy, inhibiting G9a/GLP in both early (5xFAD) [150] and late‐onset (SAMP8) [134] AD mice models result in the reversion of histone hyper‐methylation, leading to an improvement in cognitive dysfunction. In this regard, chromatin immunoprecipitation–sequencing (ChIP‐seq) data indicates that FAD mice show genome‐wide enrichment of H3K9me2 at genes involved in neuronal signaling (including glutamate receptors) [147]. Furthermore, treatment with UNC0642 in the 5xFAD mouse model reduces 5 mC levels, contributing to cognitive restoration [150] (Figure 2).

G9a inhibition decreases β‐amyloid plaques in both 5xFAD [150] and SAMP8 mice models [134]. Although the mechanism has not been fully elucidated, the reduction of Aβ load after G9a inhibition could be due to the direct modulation of non‐amyloidogenic pathway genes, leading to reduced cognitive deficits. In fact, G9a inhibitors rescue impaired recognition memory, working memory, and spatial memory in aged AD mice models [134, 147, 150]. Similarly to the effect seen in the 5xFAD mice, G9a pharmacological inhibition restores the cognitive status in SAMP8 mice [134, 150]. G9a inhibition also correlates with a decrease in anxiety‐like behaviors in adult male mice [151] and improved social behavior in the SAMP8 model [134, 152] (Figure 2).

In this regard, inhibition of G9a may prove an effective means of rescuing deficits in the long‐term potentiation (LTP) and synaptic tagging/capture in the hippocampal CA1 area of the APP/PS1 mouse model of AD by facilitating protein synthesis [153]. Likewise, G9 inhibition might promote neuronal plasticity, involving significant upregulation of nerve growth factor (*Ngf*), vascular growth factor inducible (*Vgf*), brain derivative neurotrophic factor (*Bdnf*), and synaptophysin (*Syn*) gene expression [134, 149]. Nevertheless, G9a inhibition was unable to induce cell‐wide priming and synthesis of plasticity‐related products (PRPs) in the AD‐like condition [153]. This finding contradicts previous studies in which upregulation of synaptic genes and synaptic transmission was observed in Aß‐affected cortical neurons and mouse models of AD [147]. The discrepancy could be explained by different durations of the pharmacological inhibition, as G9a activity responds rapidly to external stimuli [154] (Figure 2).

Of note, G9a pharmacological inhibition also modulates pathways related to neuroinflammation, one of the key events in AD pathogenesis. UNC0642 treatment inhibits the NF‐KB pathway and related genes such as *Il‐6, Cxcl10*, and *Tnf‐a*, leading to a neuroinflammation reduction in SAMP8 mice [134]. Accordingly, G9a inhibition decreases IBA‐1 and GFAP levels in a primary mixed culture of neurons and microglia, as well as in AD mice models [134, 150] (Figure 2).

It is noteworthy that a transcriptional profile analysis following G9a inhibition demonstrated elevated gene expression of *glia maturation factor β* (*Gmfb*) in SAMP8 mice. This finding substantiates an unidentified mechanism through which G9a inhibition may operate at two levels, namely, by increasing GMFB and regulating its function to facilitate neuroprotective effects in age‐related cognitive decline [134]. This finding agrees with several studies that show that G9a adds methyl group to a large number of non‐histone proteins, modulating their function and stability (Figure 2).

Furthermore, dysregulation of G9a correlates with increased levels of oxidative stress (OS), another factor involved in AD pathogenesis. While G9a promotes oxidative stress in neurons [93], inhibition of G9a reduces ROS levels and elevates antioxidant enzyme levels [150, 155]. Additionally, hydrogen peroxide treatment in cell cultures correlates with a pronounced increase in the repressive mark H3K9me2 [156]. UNC0642 treatment in AD mice models modulates OS responses [150, 155]. Regarding this, upregulation of PPARγ by G9a inhibition could also increase the expression of genes involved in DNA damage responses and apoptosis. In the SAMP8 mice model, G9a inhibition downregulates *miR‐128*, suggesting that the reduction of OS levels was due, at least in part, to the modulation of PPARγ‐dependent pathways (the mRNA target for this *miR‐128*) [155]. Accordingly, increases in the nuclear Factor erythroid‐2‐related factor 2 (NRF2), *Heme oxygenase decycling 1* (*Hmox1*) gene expression, and a decrease in reactive oxygen species (ROS) were also reported in the 5xFAD mice model [150, 155] (Figure 2).

Finally, it is known that G9a directly represses genes known to participate in the autophagic process and that inhibition of G9a‐mediated epigenetic repression represents an important regulatory mechanism during autophagy [157]. Hence, UNC0642 treatment activates the autophagy pathway, increasing the levels of proteins involved, such as Sequestosome‐1 (SQSTM1/p62) and Beclin‐1 (BECN1) in SAMP8 mice [155] (Figure 2).

### 6.2 Parkinson's Disease

The second most prevalent neurodegenerative disease is PD, which affects over 1% of the population aged 65 and above. It is estimated that the prevalence of this disease will double by 2030 [158, 159, 160]. PD cases can be differentiated into two forms: familial and sporadic, but the etiology of the disease remains largely unclear [161]. The familial form represents 10%–15% of cases, and the sporadic is labeled as idiopathic [162]. PD is likely to be a multifactorial disease, with genetic and environmental factors contributing to its genesis, as in the case of AD [163].

PD is characterized by both motor symptoms, such as tremors, postural instability, muscle stiffness, and bradykinesia [164], and non‐motor symptoms, including depression and cognitive dysfunction [165, 166]. At the molecular level, there is a neuronal loss in the substantia nigra, and proteinaceous aggregates composed mainly of α‐synuclein (αS) in intraneuronal inclusions [167, 168]. The loss of proteostasis, a defining feature of the aging process, may play a pivotal role in the accumulation of αS, akin to the accumulation of Aβ observed in AD brains [169] could play an important role in the accumulation of αS, similarly to what happens in the AD brain with regard to Aβ. In addition, αS deposits and their neurotoxic accumulation could be due to relative overproduction, misfolding, and oligomerization of the protein, and the presence of mutations [170, 171] like in the *αS* gene (SNCA [172], LRRK2 [173], and VPS35 [174]). Other pathways affected during disease progression include oxidative stress [175], dysfunctional mitochondria [176], cellular calcium imbalance [177, 178], neuroinflammation [179], and other neurotransmitter system deficits [180, 181].

**Figure 3:**
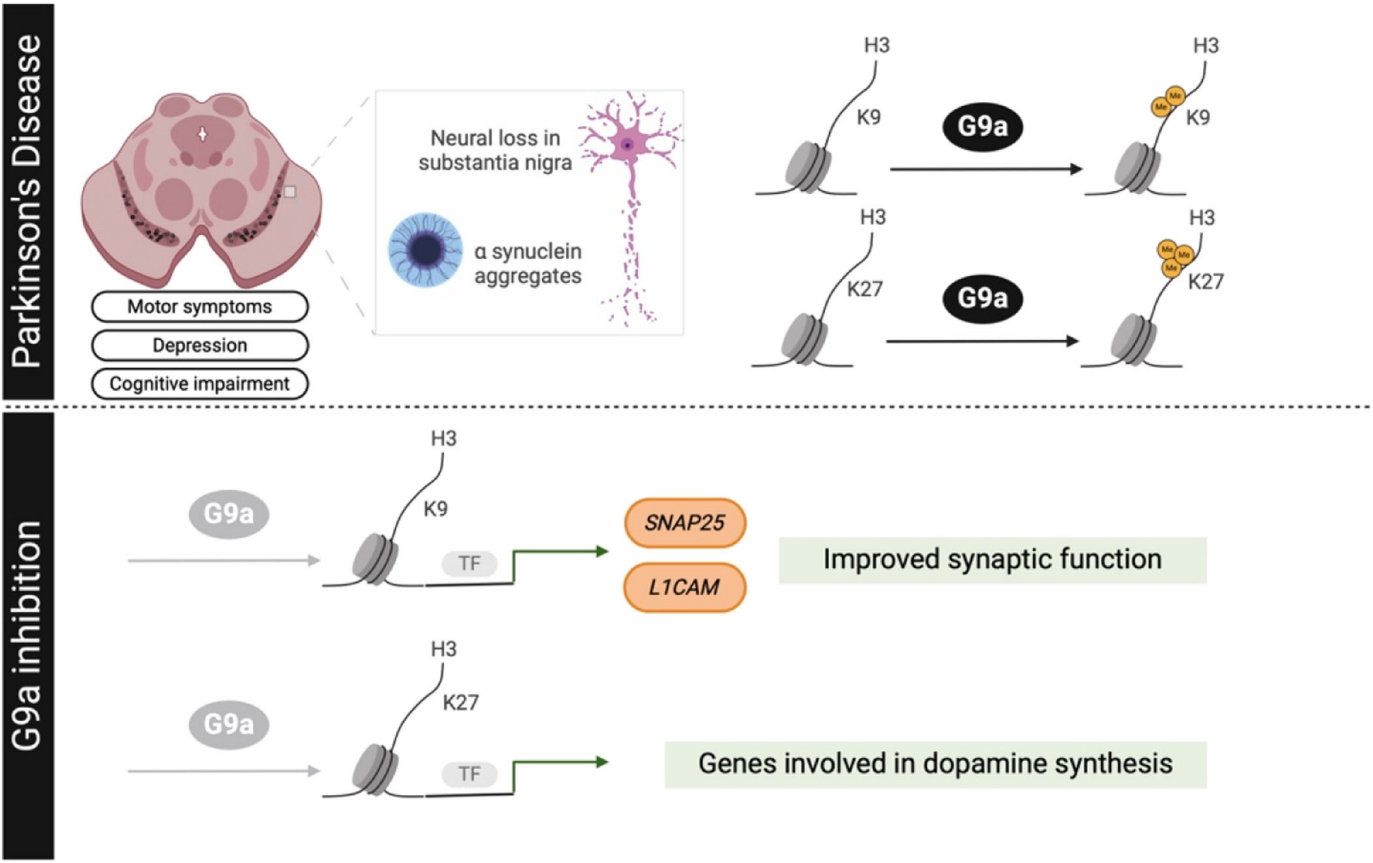
Comprehensive overview of findings in Parkinson's disease (PD) following pharmacological inhibition or genetic modification of G9a. This schematic diagram illustrates the multifaceted effects of G9a modulation on various aspects of PD pathology. [Color figure can be viewed at wileyonlinelibrary.com]

Interestingly, mutations in the *LRRK2* gene have been linked to PD pathology [173]. The genetic burden of LRRK2 variants appears to be associated with the age at the onset of the disease [182, 183, 184], and its penetrance increases with age. Indeed, *Lrrk2* deficiency disturbs genomic stability during aging, and reduced levels of H3K9me2 were found in the striatal tissues of 12‐month‐old *Lrrk2* ^*−/−*^ mice [185] (Figure 3).

In a similar manner, overexpression of αS in Drosophila and SH‐SY5Y cells leads to upregulation of the repressive marks H3K9me1 and H3K9me2, disrupting gene expression [186]. The transient increase of H3K9 methylation observed in αS‐induced SH‐SY5Y cells was preceded by induction of EHMT2 (G9a) mRNA, which suppressed the expression of *L1CAM* (the neural cell adhesion molecule L1) and *SNAP25* (the synaptosome‐associated protein). Hence, treatment with UNC0638 restored the expression levels of both genes. Furthermore, the presence of REST target genes exhibiting RE1 sites was investigated, and the repressive H3K9me2 mark within the SNAP25 promoter region was identified. This histone mark has been demonstrated to reduce gene transcription levels, which subsequently results in a decline in protein expression. This finding indicates that αS overexpression alters the distribution of histone marks in genes associated with the REST complex, resulting in impaired synaptic functions [186] (Figure 3).

Moreover, G9a is also responsible for H3K27me3 [89], which is another repressive histone mark that could lead to the silencing of genes involved in dopamine synthesis and release [187]. Additionally, G9a may also contribute to PD by promoting inflammation and OS, two of the events that play an important role in the pathology. However, the latter has only been studied in the context of AD, and it has not been described yet for PD. Therefore, further experiments are needed to assess whether these pathways are involved and their relevance during disease progression (Figure 3).

### 6.3 Huntington's Disease

HD is a hereditary neurodegenerative disorder caused by a dominantly inherited CAG repeat expansion in exon 1 of the *huntingtin* gene (*HTT*) [188]. The mutated form of the huntingtin (mHTT) protein has an expanded region of repeated CAG nucleotides in the gene [189], which leads to the production of an abnormal and toxic form of the protein known as polyglutamine (polyQ) tract. Its prevalence varies among different populations, reaching ~12 per 100,000 individuals in European populations. HD patients with longer CAG repeats have earlier symptom onset and faster disease progression since polyQ expansion toxicity is correlated with repeat size [190, 191, 192]. Motor symptoms of HD can appear from childhood to old age, with an average age of onset of about 45 years, and are followed by an inexorable progression of the disease [193, 194]. Although the HD mutation was identified in 1993, the precise mechanism by which the toxic mutant protein causes neurodegeneration remains elusive [195], and this means that for the time being there is no definitive cure for this disease [196, 197]. Therefore, it is crucial to prioritize the discovery of the molecular pathways that underlie or play a role in the development of human HD.

HTT plays a role in the development of CNS, including neural tube formation and neuroblast migration. Additionally, it is involved in axonal transport, synaptic function, and cell survival [198, 199, 200, 201]. Hence, the presence of mHTT disrupts various cellular processes, including gene expression, mitochondrial function, and axonal transport [202, 203, 204]. Over time, the accumulation of mHTT protein and the disruption of cellular processes result in neuronal damage and cell death, particularly in the striatum and other areas of the brain [205]. In fact, neurons found in the striatum, a brain part essential for movement control, are particularly vulnerable to mHTT [205, 206]. Moreover, HTT binds to DNA in numerous genes, and expanded polyglutamine tracts in HTT lead to transcriptional dysregulation [207]. Individuals with HD experience substantial alteration in transcription [208], leading to increased immune response [209] and mRNA processing [210], and decreased metabolic processes [211] and synaptic function [212] in the brain compared to healthy controls. Because of this, HD is characterized by severe motor dysfunction (chorea, abnormal involuntary movements), cognitive decline, and psychiatric symptoms [213].

**Figure 4:**
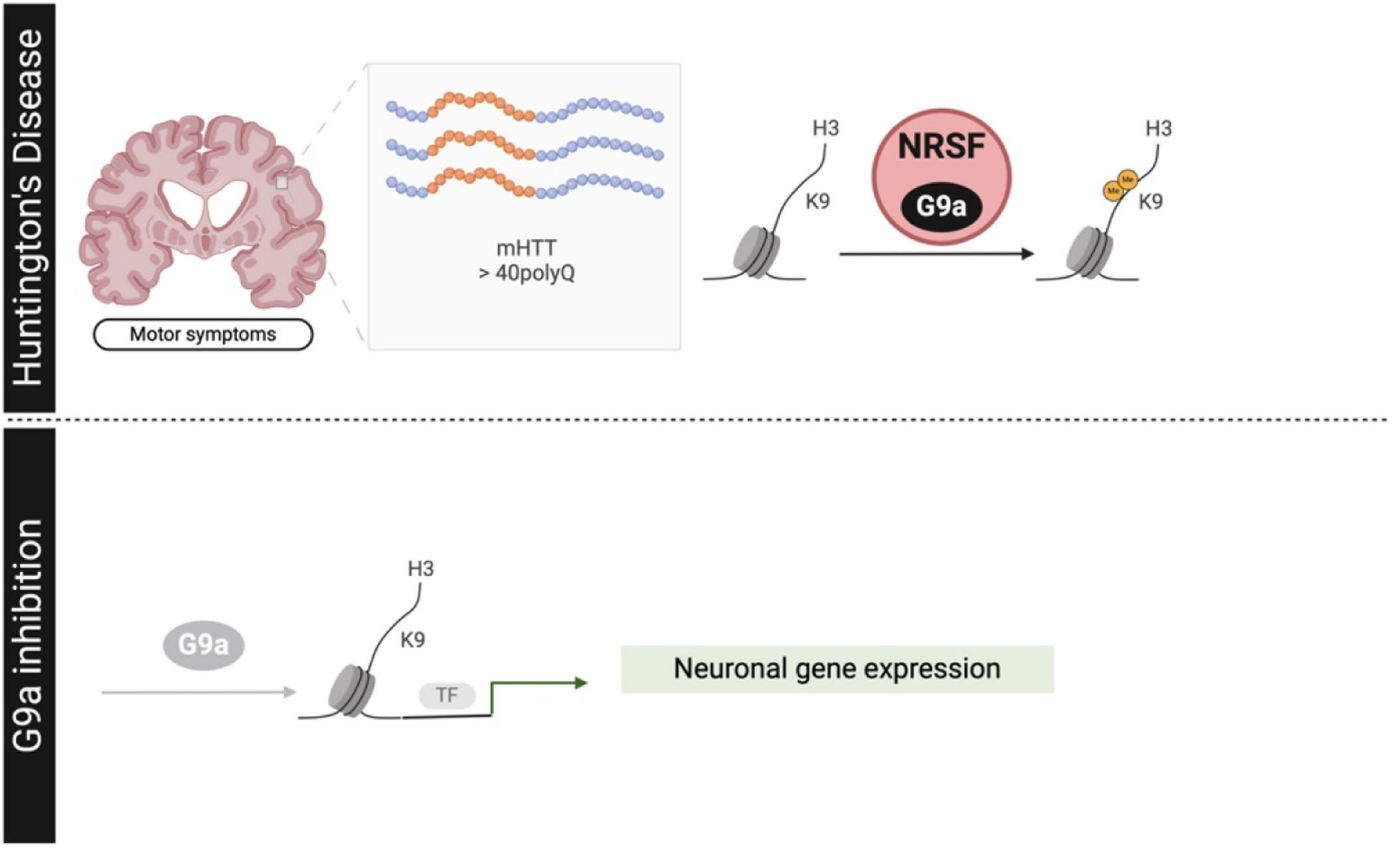
Comprehensive overview of findings in Huntington's disease (HD) following pharmacological inhibition or genetic modification of G9a. This schematic diagram illustrates the multifaceted effects of G9a modulation on various aspects of HD pathology. [Color figure can be viewed at wileyonlinelibrary.com]

While the exact molecular processes causing transcriptional dysregulation in HD are not yet fully understood, numerous studies highlight the significance of the epigenetic mechanisms [214, 215, 216, 217, 218, 219]. Advances in next‐generation sequencing technologies have improved our understanding of the HD transcriptome and epigenome. Interestingly, HD patients and various models of HD show altered patterns of several key histone marks. Methyltransferases responsible for the methylation in H3K9, and consequently the relative repressive marks (H3K9me2/3) have been observed in the striatum of both R6/2 HD mice and HD patients [220, 221, 222]. Accordingly, work studying gene expression profiles in the cortex, striatum, hippocampus, and cerebellum of juvenile R6/1 and N171‐82Q mice, models of rapidly progressive HD, concluded that one of the most prominent cases was that of conditional mutants of histone methyltransferases G9a and GLP [223]. In the same line, transcriptional repressor neuron restrictive silencing factor (NRSF, also known as REST) [224] can repress the expression of at least 30 neuronal genes outside the nervous system. In fact, NRSF recruits G9a to silence NRSF target genes in nonneuronal cells [115]. Therefore, the role of aberrant NRSF‐mediated repression in HD, among other neurological disorders, emphasizes the importance of understanding its mechanisms to regulate gene expression (Figure 4).

### 6.4 Autism Spectrum Disorder

ASD is a devastating neurodevelopmental condition with a significant epidemiological and societal impact worldwide. Currently, the prevalence of ASD is 1 in 54 children and is more common among boys than girls, according to the Centers for Disease Control [225]. The onset of ASD‐related symptoms occurs between 12 and 18 months of age, with a diagnosis typically made at ~2 years of age [226]. ASD causes are complex and multifactorial, with several associated genes and environmental risk factors [227]. Noteworthy, heritability of the disorder has been estimated to be 50%, indicating that genetic factors are the main contributors to the etiology of ASD [228].

**Figure 5:**
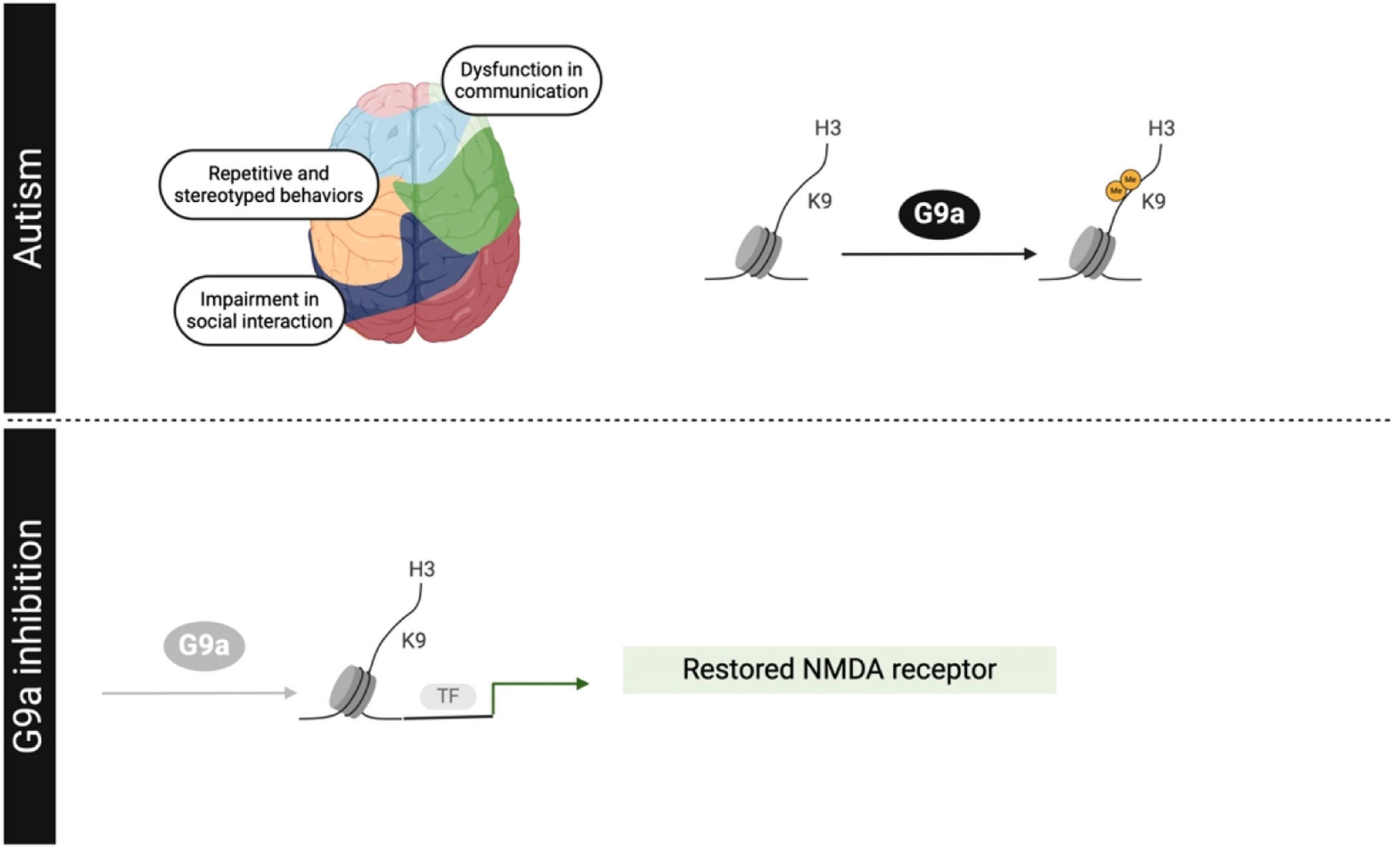
Comprehensive overview of findings in autism spectrum disorder (ASD) following pharmacological inhibition or genetic modification of G9a. This schematic diagram illustrates the multifaceted effects of G9a modulation on various aspects of ASD pathology. [Color figure can be viewed at wileyonlinelibrary.com]

On one hand, ASD is characterized by early‐onset dysfunctions in communication, impairments in social interaction, and repetitive and stereotyped behaviors and interests [229, 230]. On the other hand, at the molecular level, there are several pathways dysregulated in ASD, including chromatin remodeling, RNA transcription and splicing, synaptic function [231, 232], ion channels, MAPK, and calcium signaling [233]. It is noteworthy that a considerable number of well‐established autism risk factors are histone‐modifying enzymes that regulate histone methylation and demethylation. G9a is elevated in the blood and prefrontal cortex (PFC) of these ASD patients, suggesting an increasingly restrictive chromatin in the pathogenesis of ASD [234]. Accordingly, in *Shank3*‐deficient mice, a model of autism spectrum disorders, inhibiting G9a counteracted both elevated H3K9me2 levels and disturbed transcriptional signatures. Overall, inhibition of G9a could rescue autism‐like social deficits and restore NMDA receptor–mediated synaptic function [60]. Notably, the synaptic plasticity gene called *Arc* was described as one of the key molecules mediating the rescuing effects of Shank3‐deficient mice after G9a inhibition (Figure 5).

### 6.5 Neuropsychiatric Disorders

Schizophrenia (SZ) is a chronic and complex mental disorder that affects about 20 million people worldwide [235]. Symptoms of SZ can be categorized into three main types. The initial category of symptoms is that of positive symptoms, which encompass a distortion of typical functioning and include hallucinations, delusions, disorganized thinking, and disorganized speech. Second, negative symptoms are defined as a reduction or absence of typical functioning, including a lack of motivation, social withdrawal, and a limited range of emotions [236]. Third, SZ patients exhibit cognitive symptoms such as impaired memory, attention, and executive functioning [237]. The exact causes of SZ are unknown, but likely a combination of genetic, biological, and environmental factors contribute to its development [235]. Growing evidence suggests that SZ pathogenesis is associated with epigenetic dysregulation of genes involved in neurodevelopment, neurotransmission, and immune functions [238]. Notably, elevated levels of G9a, and consequently its repressive mark, H3K9me2, were found in postmortem PFC in SZ patients [239]. This repressive mark is associated with symptoms of SZ as well as with cognitive and learning deficits, the latter already previously discussed in the review. In this regard, the impact of the pharmacological G9a inhibition on gene expression related to SZ has been studied in peripheral blood mononuclear cells (PBMCs) from participants with SZ and in healthy control patients, as well as in an SZ mouse model [240]. BIX‐01294 treatment significantly reduced elevated H3K9me2 levels in PBMCs from SZ participants. Additionally, genes like *IL*‐6 [241], *GAD67* [242], *NANOG*, and *KLF4* [243], are commonly downregulated in SZ, and the pharmacological G9a inhibition was able to restore mRNA levels in PBMCs culture [240]. Moreover, peripheral administration of BIX‐01294 also reduced both global and promoter‐specific H3K9me2 protein levels and increased mRNA levels of SZ candidate genes such as *Reln* [244], and *Bdnf9a* [245]in a SZ mouse cortex [240].

**Figure 6:**
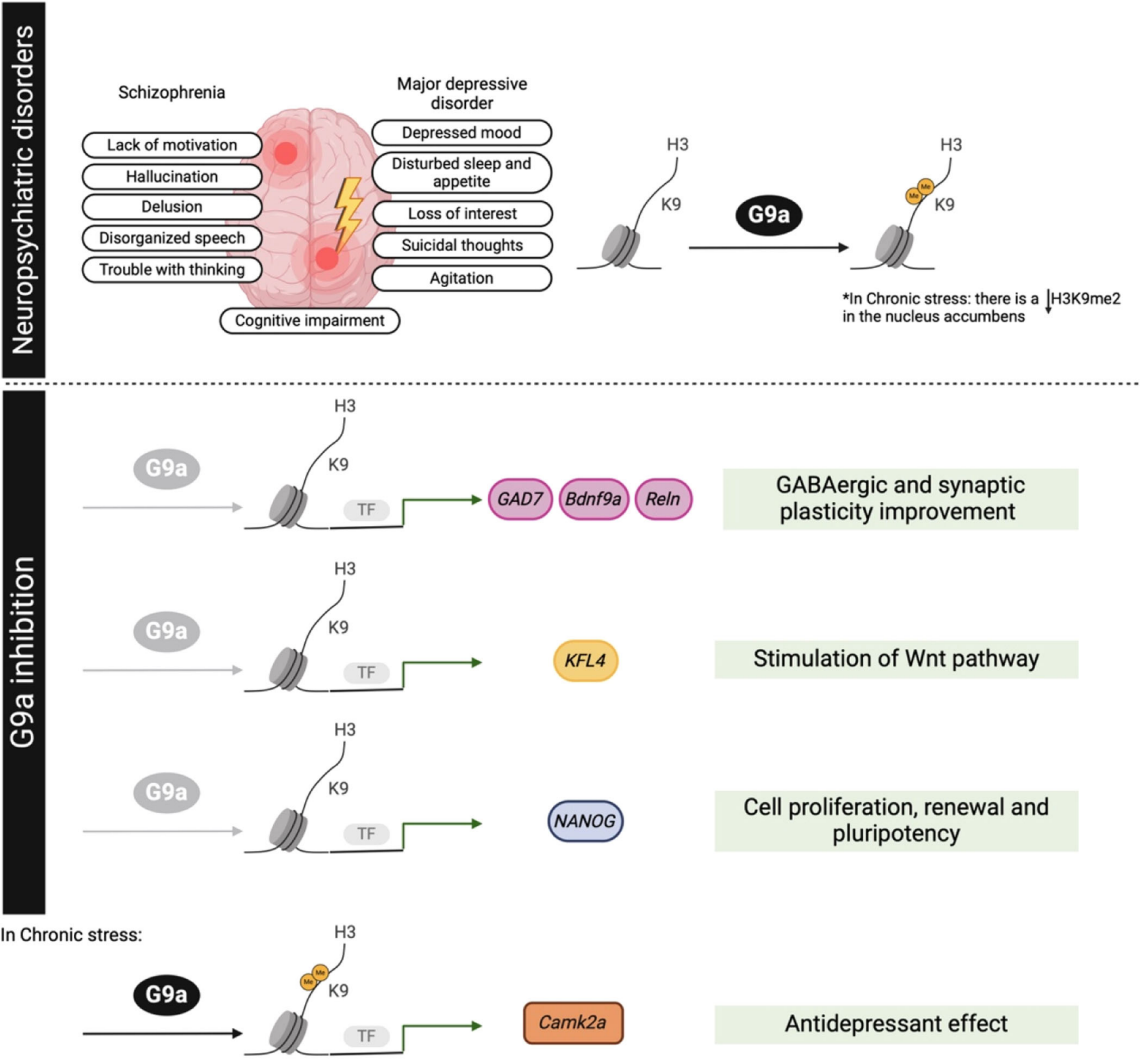
Comprehensive overview of findings in neuropsychiatric disorders following pharmacological inhibition or genetic modification of G9a. This schematic diagram illustrates the multifaceted effects of G9a modulation on various aspects of schizophrenia (SZ) and major depressive disorder (MDD) pathology. [Color figure can be viewed at wileyonlinelibrary.com]

Major depressive disorder (MDD) is a chronic, generally episodic, and debilitating neuropsychiatric disease that affects an estimated 300 million people worldwide [246]. MDD is characterized by depressed mood, disturbed sleep and appetite, loss of interest, feelings of guilt, suicidal thoughts, psychomotor retardation or agitation, and cognitive impairment [247]. Despite extensive research, the causes of MDD remain poorly understood. While heritability studies have estimated the genetic contribution to MDD at 35% [248], epidemiological studies indicate that environmental factors are also strongly associated with the risk of developing MDD and other stress‐related disorders [249, 250, 251]. Notably, recent research has directed its attention toward the epigenetic control influenced by environmental factors during early life experiences [252, 253, 254, 255]. These findings clarify how these factors may explain variations in an individual's susceptibility and resilience to stress. Specially, emerging evidence suggests that repressive histone methylation is elevated in MDD. Traumatic experiences [256] and chronic unpredicted mild stress (CUMS) [257] lead to an increase in H3K9me2 at the *Bdnf* promoter, reducing its expression in the prefrontal cortex (PFC), hippocampus, and the amygdala, resulting in dendrite maldevelopment and a higher risk of mental disorders than in the general population, categorized as posttraumatic stress disorder (PTSD) [256, 257]. In this regard, since pharmacological inhibition of G9a reduces H3K9me2, depressive behavior was alleviated in adolescent and adult rats [258]. By contrast, chronic stress has also been found to reduce overall levels of H3K9 di‐methylation (H3K9me2), accompanied by a decrease in the expression of the enzyme G9a [259]. This is because activation of G9a leads to increased H3K9me2 in the *Camk2a* gene, a gene crucial for synaptic plasticity, inducing antidepressant effects [259] (Figure 6). Thus, research suggests that histone methylation might have adaptive effects in stress models.

## 7 G9a Inhibitors

### 7.1 Rational Design and Synthesis of G9a Inhibitors

In 2007, Kubicek et al. [260] shortlisted 7 hits from the preselected chemical library containing 125,000 compounds. Among them, **BIX‐01294** (**1**), a diazepinquinazolin‐amine derivatives, emerged as a selective inhibitor of G9a, a histone methyltransferase (HTMase) along with noncompetitive cofactor S‐adenosyl‐methionine (SAM) with IC_50_ of 2.7 µM in dissociation enhancement lanthanide fluro‐immuno assay (DELFIA). Moreover, Chang et al. [261] published the crystal structure analysis of (**1**) and *S*‐adenosyl‐l‐homocysteine with GLP's catalytic SET region (PDB ID: 2RFI) in which ligand bound with substrate peptide groove positioned near lysine residues, reporting an IC_50_ of 0.7 µM against GLP.

**Figure 7:**
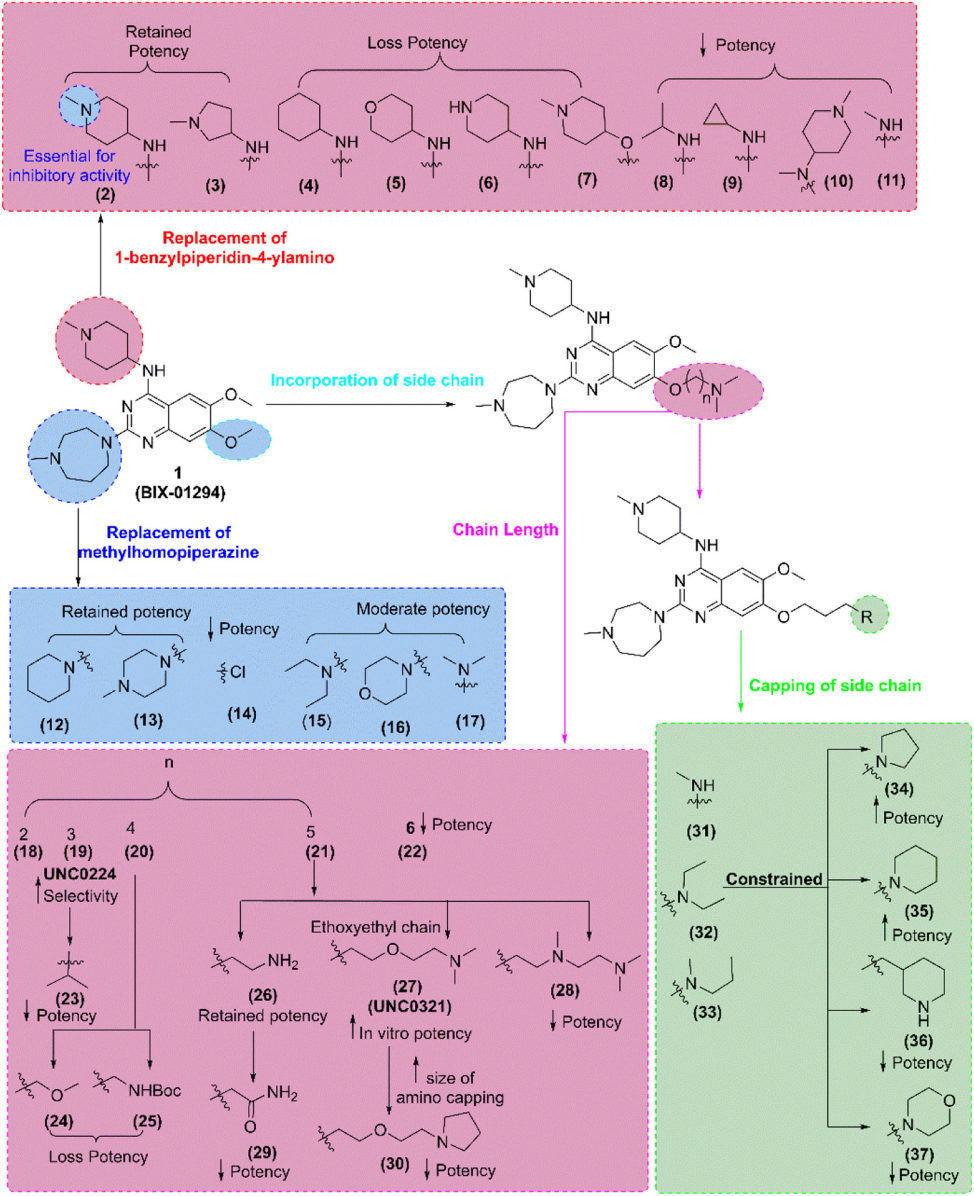
Rational design and synthesis of G9a inhibitors (Part I): Schematic illustration of the structure–activity relationship (SAR) studies and key modifications leading to the development of potent G9a inhibitors. [Color figure can be viewed at wileyonlinelibrary.com]

Then, Liu et al. [262, 263] described the structure–activity relationship (SAR) (Figure 7) and discovered and synthesized a novel 2,4‐diamino‐7‐aminoalloxyquinazoline as an HTMase G9a inhibitor (**19**/**UNC0224**). First, authors biologically evaluated the synthesized compounds by ThiaGlo‐based G9a inhibitory assay or enzyme‐coupled SAH detection (ECSD) assay and G9a AlphaScreen assay or chemiluminescence‐based oxygen tunneling (CLOT) (Supporting Information S7: Table S1) which used for conversion of SAM to *S*‐adenosyl‐l‐homocysteine (SAH) monitoring, and methylated histone peptides detection, respectively. They designed different compounds (**2–11**) by substituting the 1‐benzylpiperidin‐4‐ylamino group (**1**), and this allowed the authors to conclude that secondary nitrogen is involved in the hydrogen bonding interaction with the amino acid Asp1083 of the G9a enzyme to inhibit G9a activity. In the case of 2‐amino moiety within the quinazoline scaffold, alterations to the 2‐amino (**1**) region were well tolerated (**12–17**) (Figure 7). Authors evaluated the effect of carbon numbers in the aminoalkoxy side chain on the potency of G9a and found that carbon numbers 2–5 (**18–21**) maintained the potency and carbon chain length up to 6 (**22**) decreased the potency, which indicated that 5‐carbon number in aminoalkoxy side chain easily occupied in the lysine binding region of G9a to increase the potency and selectivity because nitrogen atom in side chain interacts with Leu1086 and Tyr1154 via electrostatic and π‐cation bond. Among them, compound (**19**) showed improved potency along with selectivity for G9a. For evaluation of the effect of the nitrogen‐contained side chain of aminoalkoxy, authors incorporated methine (**23**) instead of 7‐dimethylamino propoxy substituent of (**19**) and found that it decreased the potency 100 times due to the absence of interaction with the lysine residue of G9a enzyme.

**Figure 8:**
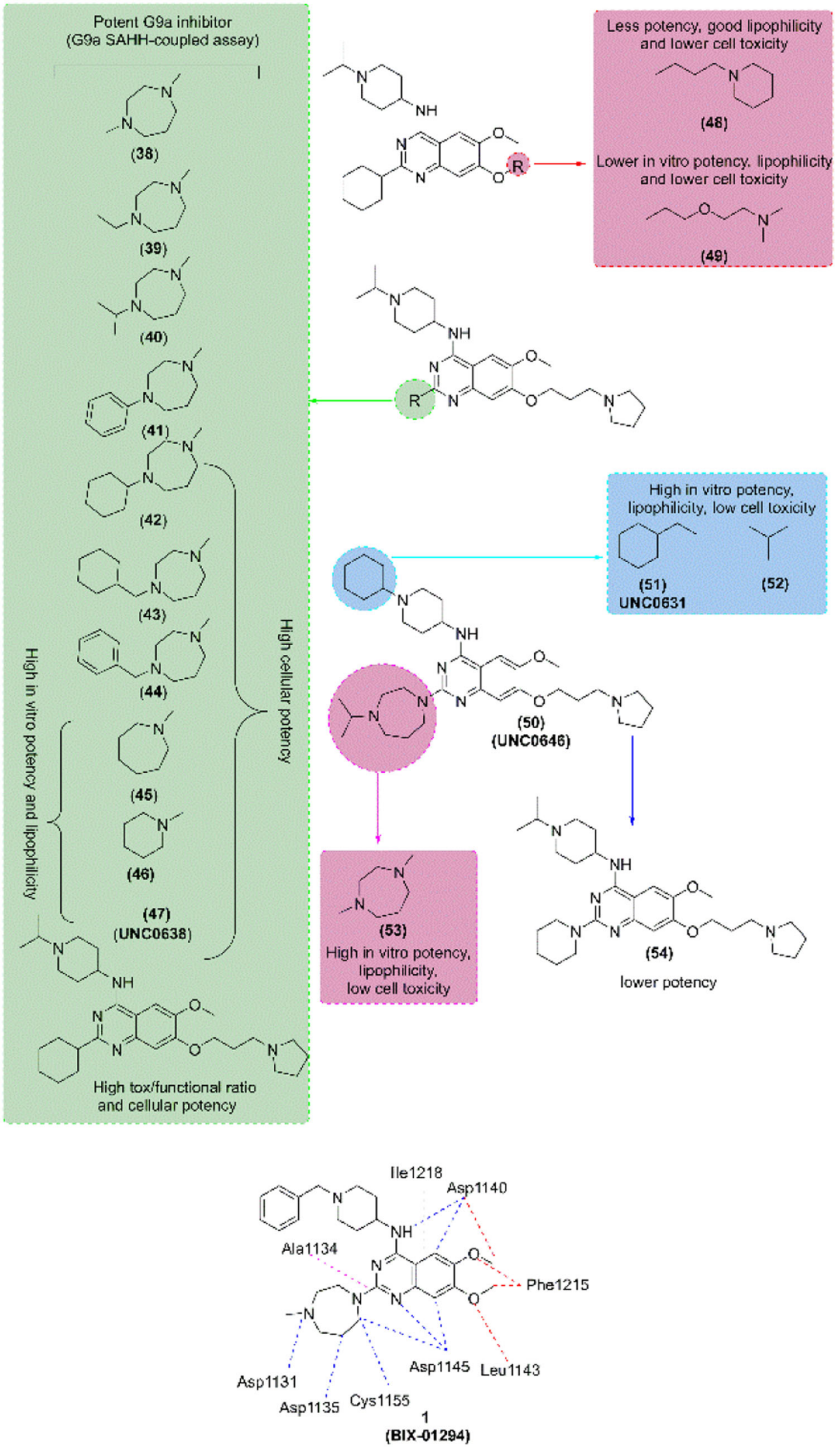
Rational design and synthesis of G9a inhibitors (Part II): Schematic illustration of further structure–activity relationship (SAR) studies and key modifications leading to the development of more potent and selective G9a inhibitors. [Color figure can be viewed at wileyonlinelibrary.com]

**Figure 9:**
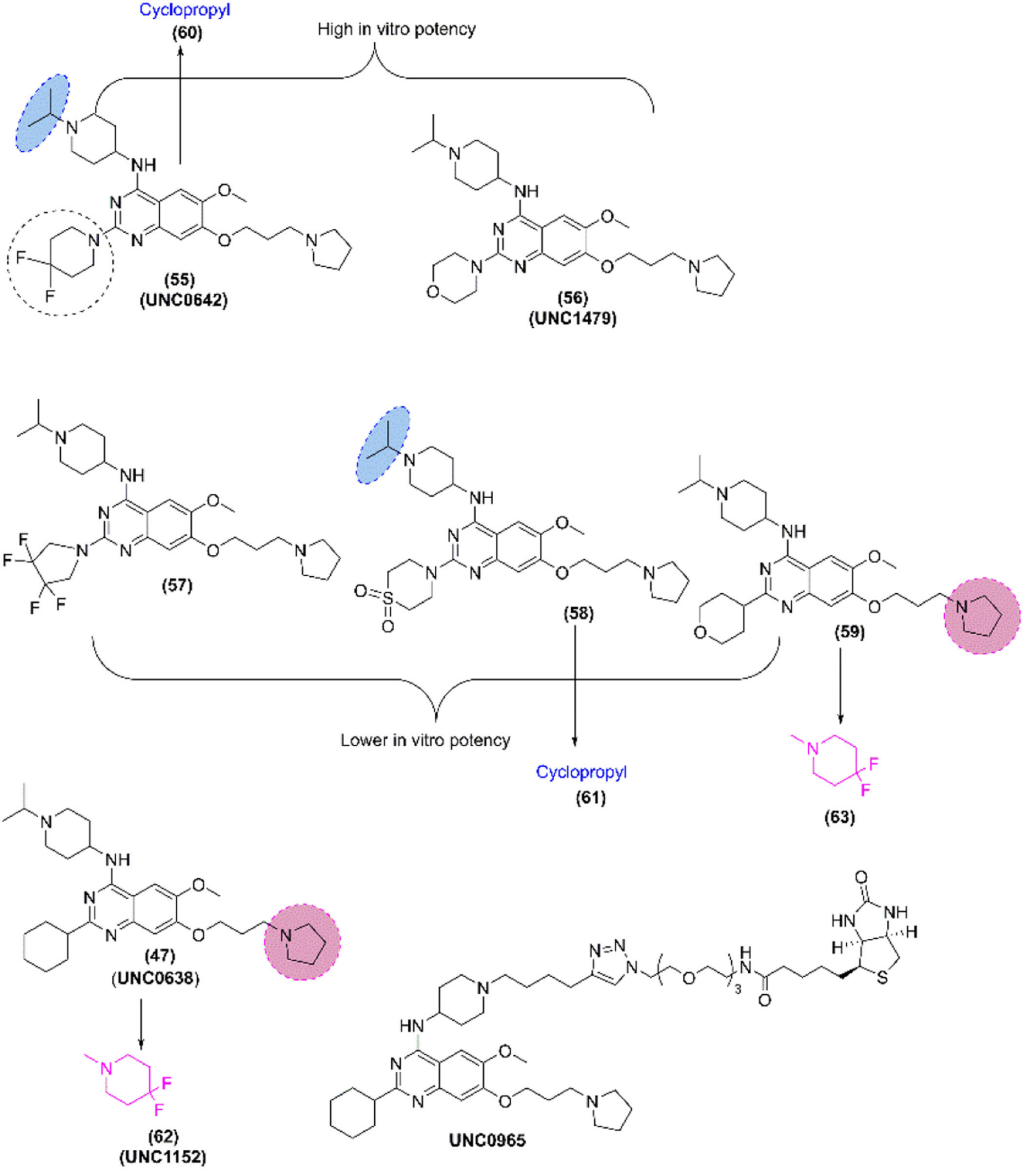
Rational design and synthesis of G9a inhibitors (Part III): Schematic illustration of further structure–activity relationship (SAR) studies and key modifications leading to the development of more potent and selective G9a inhibitors. [Color figure can be viewed at wileyonlinelibrary.com]

Likewise, Chang et al. [264] reported a potent and less toxic G9a inhibitor, **E72**; however, its cellular potency is lower than that of (**1**). In 2011, Liu et al. [265, 266] optimized 7‐aminoalkoxy‐quinazoline (Figure 8) and evaluated the G9a inhibitory activity via G9a *S*‐adenosyl‐l‐homocysteine hydrolase (SAHH)‐coupled assay, cellular potency via In‐Cell Western (ICW) assay, and cellular toxicity via 3‐(4,5‐dimethylthiazol‐2‐yl)‐> 2,5‐diphenyl tetrazolium (MTT) assay (Supporting Information S8: Table S2). Authors evaluated the effect of various 2‐amino group of pyrrolidine‐1‐ylprooxy derivative (**38–47**) and found that all derivatives showed high G9a inhibitory potency in G9a SAHH‐coupled assay in which compound (**45–‐47**) showed high in vitro potency along with improved lipophilicity while (**41–47**) showed high cellular potency and lipophilicity. Besides, they evaluated the combined compound encompassing different 4‐ and 2‐amino groups of pyrrolidine analog where compound (**50–53**) showed improved lipophilicity, in vitro and cellular potency, and low cellular toxicity while compound (**54)** decreased the potency. Among them, compounds (**50/UNC0646**) and (**51/UNC0631**) [265] showed high potency and tox/functional ratio but higher toxicity than (**47**). Later, [267], incorporated modifications in (**47**), and it was found that 2‐(4,4‐difluoropiperidin‐1‐yl) (**55/UNC0642**), 2‐(morpholin‐4‐yl) (**56/UNC1479**) were able to retain the in vitro potency; however, few modifications were tolerable (Figure 9). Moreover, Yuan et al. and Sweis et al. [268, 269] independently reported **BRD9539** and **A366** as potent and selective G9a inhibitors in 2012 and 2014, respectively.

In 2014, Konze et al. [270] designed and synthesized (**64/UNC0965**) as a biochemical characterization chemical tool, which is the biotinylated version of **UNC0638** to facilitate the precipitation of G9a from whole cell lysate. Ma et al. [271] discovered (**65/UNC0379**) as a substrate competitive inhibitor of lysine methyltransferase called SETD8 with selectivity over 15 other methyltransferases (Figure 9). Moreover, Srimongkolpithak et al. [272] performed various studies to improve G9a inhibitory activity with knowledge of pharmacophoric key features of the **BIX‐01294** compound, and they found new quinazoline derivatives such as **HKMTI‐1‐005**, **HKMTI‐1‐022**, and **HKMTI‐1‐011** (**89–91**) (Supporting Information S1: Figures S1 and Supporting Information S2: Figure S2). Recently, Xiong et al. [273] evaluated the inhibitory activity of GLP and G9a of different derivatives of quinazoline where they evaluated the effect of various 2‐amino groups, *N*‐capping groups of piperidine ring, 6 and 7 methoxy substituents of quinazoline, and quinoline scaffold and drew the SAR of quinazoline derivatives (Supporting Information S3: Figure 3, Supporting Information S9: Table S3). Furthermore, Charles et al. [274] performed high‐throughput screening to discover a novel quinoline‐based G9a inhibitor called **CSV0C018875** (Supporting Information S4: Figure S4).

In summary, the following table details the most important compounds during the above process (Table 2).

**Table 2:** Selection of the most commonly used G9a inhibitors (NR, non‐reported).

Compound	Derivative from	Type	IC_50_ GLP	IC_50_ G9a	Effects reported in	Reference
BIX‐01294		Substrate competitive inhibitors	38 µM	1.7 µM	Cancer, AD, malaria	[147, 260, 275, 276]
UNC0224	BIX‐01294	Substrate competitive inhibitors	50 nM	15 nM	Cancer, malaria	[265, 275]
UNC0638	BIX‐01294	Substrate competitive inhibitors	19 nM	< 15 nM	Cancer, AD, PD malaria	[17, 186, 266, 275]
E72	BIX‐01294	Substrate competitive inhibitors	0.1 µM	NR	NR	[264]
UNC0321	UNC0224	Substrate competitive inhibitors	15 nM	9 nM	Cancer, diabetic vascular complication, malaria	[263, 277, 278]
UNC0646	UNC0638	Substrate competitive inhibitors	NR	6 nM	Cancer, malaria	[265, 275, 279]
UNC0642	UNC0638	Substrate competitive inhibitors	3.7 nM	< 2.5 nM	Cancer, AD, malaria, PTSD	[134, 147, 150, 258, 266, 275, 280, 281]
UN0631	UNC0646	Substrate competitive inhibitors	NR	4 nM	Cancer, malaria	[265, 275]
MS012	BIX‐01294, E72, UNC0638	Substrate competitive inhibitors	7 ± 2 nM	992 ± 337 nM	NR	[273]
M0124	BIX‐01294, E72, UNC0638	Substrate competitive inhibitors	13 ± 4 nM	440 ± 63 nM	NR	[273]
A366		Substrate competitive inhibitors	38 nM	3.3 nM	Cancer	[268]
HKMTI‐1‐247		Substrate competitive inhibitors	NR	13 ± 1 nM	NR	[272]
HKMTI‐1‐248		Substrate competitive inhibitors	NR	31 ± 3 nM	NR	[272]
CM‐272		Substrate competitive inhibitors	NR	8 nM	Cancer	[282]
BRD9359		SAM competitive inhibitors	NR	6.3 µM	Cancer	[269]

### 7.2 New G9a Inhibitors via Advanced Computational Methods

**Figure 10:**
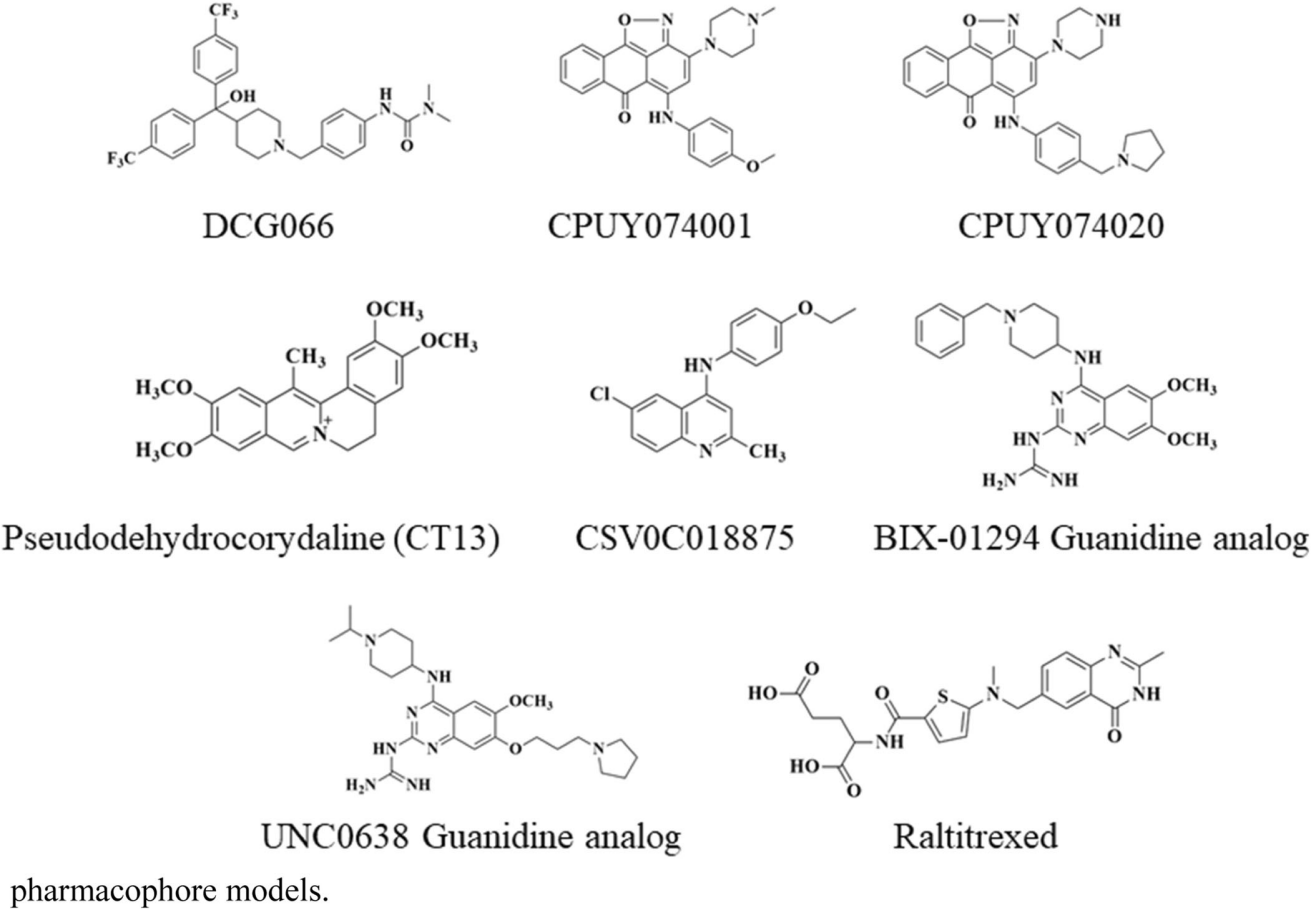
New G9a inhibitors discovered via computational methods.

Many new scaffolds have recently been discovered using advanced computational methods such as virtual screening, 3D‐QSAR, and pharmacophore mapping. In 2016, Kondengaden et al. [283] discovered DCG066, as a novel G9a inhibitor through structure‐based virtual screening. It can successfully stop leukemia cells from growing, as they express more G9a than normal cells that are not very cytotoxic. In K562 cells, DCG066 was shown to have an IC_50_ value of 1.7 ± 0.1 μM, and it can lower the histone H3 lysine 9 di‐methylation (H3K9Me2) levels like BIX01294 [283]. In another study of the shape‐based pharmacophore model using “ROCS” (Rapid Overlay of Chemical Structure), a virtual screening platform of OMEGA was developed by Chen et al. [284], and UNC0638 was employed as a reference. With a 6H‐anthra[1,9‐cd] isoxazol‐6‐one chemical scaffold, CPUY074001 was discovered with encouraging inhibitory actions against G9a (IC_50_: 39.19 ± 0.73 μM). Following that, two series of compounds were created and synthesized using a 3D quantitative structure–activity relationship (3D‐QSAR) based on the structures of CPUY074001 and G9a. Several of these chemical scaffolds have strong G9a inhibitory action. Among them, CPUY074020 was chosen for more studies because it can lower the expression of H3K9me2, in addition to having strong G9a inhibitory action (IC_50_: 2.18 ± 0.013 μM) and antiproliferative activity against tumor cell lines. It was noteworthy that CPUY074020 showed somewhat higher activity than UNC0638 against the HCT116 and MCF‐7 cell lines. In 2018, Chen et al. identified the protoberberine alkaloid, pseudodehydrocorydaline (CT13) as a novel G9a/EHMT2 inhibitor, through structure‐based virtual screening of an internal library of natural product chemicals [109, 259, 260, 261, 285, 310]. CT13 demonstrated selectivity over other methyltransferases including EZH2, SET7/9, PRMT5, and PRMT3. In studies using cells, CT13's activity level was comparable to that of BIX‐01294. Medina‐Franco and his collaborators investigated the structure–activity relationship (SAR) of G9a inhibitors using activity landscape modeling [286, 287]. Using SAR studies, important substituents linked to the inhibitors’ selectivity and effectiveness, as well as important protein–ligand interactions that propel G9a inhibition, have been found. The significance of the interactions between the lysine mimetic substituents and G9a's residue, like Asp1083, Leu1086, Asp1088, Tyr1154, and Phe1158, was further highlighted [288]. Charles et al. [289] discovered CSV0C018875, a strong and novel G9a inhibitor with noticeable inhibitory action in both enzymatic and cell‐based in vitro tests using a high‐throughput virtual screening method. The derivative's ability to inhibit G9a was demonstrated by molecular docking and dynamics studies, even in the absence of a lysine mimic side chain, which is essential for G9a inhibition. This is because the 4‐ethoxyanilino moiety was inserted deeper into the lysine tunnel, which led to the formation of a tight complex [289]. Moreover, Charles et al. [290] demonstrated the structural need of Arg8 for effective binding of the H3 substrate peptide to G9a using molecular dynamics simulations of five distinct mutant or altered G9a substrate peptides. Based on the rationale, they introduced the guanidine group (side chain of Arg) to BIX‐01294 and UNC0638, two recognized G9a inhibitors, to imitate the Arg8 of the H3 substrate peptide. The G9a binding capacity to the newly created guanidine analogs BIX‐01294 and UNC0638 was assessed by docking, DFT, and (steered) molecular dynamics simulation tests. The binding energy calculations using MMPBSA and LIE demonstrate a noteworthy enhancement in binding for the guanidine analogs in contrast to their original ligands [291]. In 2022, Jana et al. [292] developed a 3D QSAR pharmacophore model based on the validated G9a inhibitor to perform a virtual screening approach from a natural product database. They pointed out raltitrexed, a side‐line anticancer drug, as the final lead inhibitor. They reported the G9a/EHMT2 inhibitory effectiveness and anti‐Alzheimer's potential of raltitrexed. Jana et al. [293] reported an intergraded ligand and structure‐based pharmacophore model based on the available G9a‐inhibitors crystal structures. They discovered three virtual lead inhibitors employing the blended pharmacophore models (Figure 10).

### 7.3 Dual Inhibitors

Zang et al. [294] discovered dual inhibitors of G9a and HDAC, in which compound **171** showed the most promising cellular potency for both G9a and HDAC8 with G9a IC_50_ = 7.136 µM (Supporting Information S5: Figure S5). Moreover, they [295] also designed and synthesized quinazoline‐based hydroxamic acid derivatives, which produce inhibitory activity against GLP and HDAC (Supporting Information S10: Table S4). In parallel fashion, San José‐Enériz et al. [282] discovered a novel dual inhibitor of G9a and DNA methyltransferase (DNMTs) with selectivity where the author found compound **CM‐272** showed the highest G9a/DNMT inhibitory activity with IC_50_ for G9a = 8 nM and 382 nM for DNMT1. It stimulated apoptosis and inhibited cell proliferation to cause cell death. Then, Rabal et al. [296] described their SAR where they modified the quinoline ring's 4, 6, and 7‐position, and found that any modification in the methoxy group with a small group positioned at the 6‐position of the quinoline ring decreases the DNMT1 and G9a activity and modulates the compounds' ADME profile (Supporting Information S6: Figure S6). Later, the same group [297] designed and synthesized novel quinoline‐based epigenetic inhibitors, which showed potency towards three targets, G9a, DNMT1, and HDAC1/6, and described the different substitution effects on the activity (Supporting Information S4: Figure S4).

Another dual approach is G9a/EZH2 developed for several cancers, including multiple myeloma (MM) and acute myeloide leukemia (AML) [298, 299]. Research suggests that inhibitions of both G9a and EZH2 can suppress cancer cell proliferation by regulating various signaling pathways and promoting the differentiation of cancer cells. For instance, the study found that the addition of the dual inhibitor **HKMTI‐1‐005** to ATRA (all‐trans retinoic acid) is a promising therapeutic approach. **HKMTI‐1‐005** showed an inhibitory activity with IC_50_ range from 2 to 10 µM.

### 7.4 G9a Inhibitors as a Promising Treatment for Alzheimer's Disease

On the one hand, the first in vivo proof of concept (PoC) was “inhibition of EHMT1/2 rescues synaptic and cognitive functions for Alzheimer's disease” [147] using BIX‐01294 as a G9a inhibitor. As previously described, the findings reported in this article suggested that this pharmacological approach was a potential therapeutic strategy for Alzheimer's disease. On the other hand, the second in vivo PoC was developed by Griñán‐Ferré et al. in 2019 [150]. This study investigated the neuroprotective effects of the G9a/GLP complex inhibition by UNC0642 in a transgenic mouse model of EOAD.

Sbardella and colleagues developed in 2019 [300] EML741, which is a potent G9a/GLP inhibitor with high in vitro and cellular potency. The IC_50_ value for G9a is 23 nM, while the *K*_d_ value is 1.13 μM. Likewise, EML741 has been demonstrated to inhibit DNMT1 with an IC_50_ of 3.1 μM, while exhibiting no effect on DNMT3a or DNMT3b. Furthermore, it exhibits low cell toxicity, is membrane‐permeable, and can penetrate the blood–brain barrier (BBB). EML741 has demonstrated enhanced inhibitory potency against DNA methyltransferase 1 and improved selectivity against other methyltransferases. The co‐crystal structure of GLP in complex with EML741 provides a basis for the further development of benzodiazepine‐based G9a/GLP inhibitors. EML741 shows promising potential as a G9a/GLP inhibitor with favorable properties for further development and potential therapeutic applications.

A study published in 2022 [17] used structure‐based virtual screening to identify potential G9a inhibitors, followed by in vitro and in vivo analyses. The identified G9a inhibitors showed promise in reducing age‐dependent paralysis and targeting G9a by reducing H3K9me2, a histone modification associated with gene repression in learning and memory. The study also assessed the BBB permeability and impact on amyloid‐β aggregation, providing promising results for the development of G9a inhibitors as anti‐Alzheimer's agents [17].

Moreover, a significant finding is the repurposing of raltitrexed as an effective G9a inhibitor and a promising anti‐Alzheimer's agent. Raltitrexed inhibits G9a and lowers the levels of H3K9me2, suggesting its potential as an anti‐Alzheimer's therapy [15].

A recent preprint reported the development of a novel brain‐penetrant inhibitor of G9a, MS1262, which was found to block the G9a‐regulated proteolytic mechanism associated with Alzheimer's disease. The intermittent treatment with MS1262 consistently restored both cognitive and noncognitive functions to healthy levels in multiple AD mouse models, indicating its potential as a neuroprotective agent [301].

These studies highlight the potential of G9a inhibitors in the context of AD treatment. The findings offer valuable insights into the development of novel therapeutic strategies targeting G9a for the management of this neurodegenerative condition. Further research and clinical investigations are warranted to advance the potential use of G9a inhibitors as anti‐Alzheimer's agents.

### 7.5 Status on the Basic and Clinical Trials of G9a Inhibitors

The development of G9a drugs is still in its infancy. The first G9a/GLP inhibitor BIX‐01294 lowers the amounts of di‐methylated H3K9 in several G9a target genes, encouraging autophagy‐dependent cell death and lowering cell division in several cancer cell lines, including glioma, colorectal, breast, and bladder cancer [260, 302, 303]. At concentrations exceeding 4.1 μM, BIX01294 was observed to exert a detrimental effect on cellular viability. The utilization of the compound as a G9a chemical probe has been restricted by the weak separation between the concentration providing strong functional effects in cells and the concentration causing toxicity [304]. UNC0224, which was one of the first enhanced G9a probes after BIX‐01294, was noted in 2009 to exhibit notable improvements in G9a potency [305]. Later research on the quinazoline UNC0224 led to the identification of the more effective and focused G9a/GLP probe molecule UNC0321 [306]. These probe molecules had not yet been examined for cellular potency or indications of target interaction inside a cellular setting. Following further molecular optimization, UNC0638 was synthesized and showed strong efficacy and specificity for G9a, along with increased lipophilic qualities, cell membrane permeability, and reduced cell toxicity [307]. UNC0638 has demonstrated efficacy in vitro, effectively inhibiting the growth of cells in a range of cancer cell lines, including those from the breast, squamous head and neck, hepatocellular, acute myeloid leukemia, and cervical cancer [307]. UNC0642, a G9a and GLP inhibitor suited for animal investigations, was synthesized by Liu et al. The compound was proven to have low cell toxicity and great selectivity while exhibiting enhanced pharmacokinetics as compared to other chemical probes. Jiayang YH and his collaborators found that in both the PWS mouse model and the human PWS patient‐derived cells, UNC0638 and UNC0462 may reactivate the crucial PWS genes from the maternal chromosome [308, 309]. Normal wild‐type mice did not exhibit any substantial harm from the medication, but it might reverse the perinatal death associated with the PWS mouse model. The findings offer evidence in favor of investigating the first epigenetic treatment in PWS by altering the epigenetic state [308, 309]. By affixing lysine mimic groups to the BIX01294 scaffold, compound E72 was created using a somewhat different method. In assays based on enzymes, the chemical exhibited greater activity than BIX01294, but in studies based on cells, its ability to lower H3K9me2 levels was not as strong [310]. E72 has been demonstrated to prevent the proliferation of human colon cancer cells (RKO) and reactivate K‐ras–mediated epigenetic silencing of the Fas gene in NIH 3T3 cells [310]. Another class of H3 peptide competitive inhibitor of G9a was also discovered, and it was based on the indole core molecule A‐366, which has a greater selectivity for G9a than GLP [311]. Using xenograft models of leukemia, A‐366 has been shown to exert pro‐differentiation effects on leukemia cell lines, sensitivity to DNA double‐strand break inducers in osteosarcoma, and reduce tumor burden in vivo [311, 312]. The possibility of differentiation treatment in hepatocellular cancer was highlighted by the recent suggestion that the new medication CM‐272, a dual G9a/DNA methyltransferase (DNMT) inhibitor, might promote hematopoietic stem cell differentiation and growth inhibition [313]. In both in vitro and animal models of hematological malignancies, bladder, hepatocellular carcinomas (HCCs), and hematological malignancies, CM‐272 has shown significant therapeutic effectiveness with decreased toxicity [314, 315, 316]. In addition to reducing global DNA methylation and H3K9me2 levels, CM‐272 also demonstrated the capacity to decrease cell viability and proliferation while inducing apoptosis. The medication slowed the growth of tumors and hindered the course of the illness when applied in vivo [314, 315, 316].

Recently, Park, K.S. et al. [317] discovered MS8511, the first‐in‐class G9a/GLP covalent irreversible inhibitor focusing on a cysteine residue at the substrate binding site. MS8511 exhibited a two‐fold enhancement in binding affinity to G9a, as evidenced by an ITC biophysical assay, and a five‐fold increase in potency in the inhibition of G9a methyltransferase activity, as demonstrated by an SAHH‐coupled biochemical assay in comparison to UNC0642. However, MS8511 still exhibited comparable binding affinity and potency for GLP. MS8511 showed better antiproliferation action and reduced H3K9me2 level in cellular tests when compared to UNC0642 [317]. In 2023, Feng et al. [318] used the structure‐based drug design technique and SAR research, and a very effective and selective covalent inhibitor, Compound 27 of G9a/GLP, was found [318]. Compound 27 successfully decreased the amounts of H3K9me2 in cells in a manner that was dependent on both dosage and time. Moreover, 27 demonstrated notable anticancer activity in the PANC‐1 xenograft model without causing a noticeable weight loss or toxicity [318]. Nishigaya et al. [319] reported a structurally unique and strong G9a/GLP inhibitor, RK‐701. RK‐701 demonstrated notable specificity towards other homologous methyltransferases, as well as dose‐dependent reduction of cellular H3K9me2 concentrations and suppression of tumor development in MOLT‐4 cells in vitro. In a carcinogen‐induced HCC in vivo mouse model, RK‐701 demonstrated a reduction of tumor development and progression without overt acute toxicity [319].

## 8 Conclusions and Future Directions of G9a Inhibitors for CNS Conditions

This comprehensive review explores the role of G9a, a histone lysine methyltransferase, in neurodegenerative conditions and its potential as a therapeutic target. The review covers the structure, discovery, and function of G9a, its involvement in several neurodegenerative diseases, and the development of G9a inhibitors. Key findings highlight G9a's role in dementia, autism spectrum disorder, and neuropsychiatric disorders. The review also delves into the design, synthesis, and structure–activity relationships of G9a inhibitors, including dual inhibitor approaches targeting G9a along with other epigenetic enzymes. However, while G9a inhibitors show significant potential as anticancer agents, especially for CNS tumors, more research is needed to optimize their efficacy, selectivity, and safety before they can advance to clinical trials. The focus should be on developing context‐specific inhibitors and evaluating combination therapies to maximize therapeutic benefits while minimizing potential side effects. Thus, recent developments in G9a inhibitors, such as EML741 and MS1262, and the repurposing of raltitrexed show promise for treating neurodegenerative conditions, particularly AD. The authors emphasize the potential of G9a inhibitors as a novel therapeutic strategy and call for further research and clinical investigations to advance their use in treating neurodegenerative disorders.

## Supporting information

Supporting information.

Supporting information.

Supporting information.

Supporting information.

Supporting information.

Supporting information.

Supporting information.

Supporting information.

Supporting information.

Supporting information.

## Data Availability

Data sharing does not apply to this review, as no data sets were generated or analyzed during the current study, and the supplementary figures and tables of this review are available in the Supplementary Material.
