# Supplementary material for: G9a an Epigenetic Therapeutic Strategy for Neurodegenerative Conditions: From Target Discovery to Clinical Trials
Source: Med Res Rev. 2025 Jan 6;45(3):985–1015. doi: 10.1002/med.22096 (PMC11976383; doi:10.1002/med.22096)
Supplement: Supplementary file 7 — Supporting information. [file MED-45-985-s005.docx]

**Supplementary Table 1.** G9a inhibitory activity of quinazoline derivatives via different assays.

| **Compounds** | **G9a IC_50_ (µM)** | | **G9a IC_50_ (nM)** | |
| --- | --- | --- | --- | --- |
|  | **Thioglo assay** | **Alpha-Screen** | **ECSD** | **CLOT** |
| **1/BIX-01294** | 0.11 | 0.29 | 180 | 250 |
| **2** | 0.33 | 0.23 | 330 | 230 |
| **3** |  |  | 910 | 1900 |
| **4** | <30% inhibition at 1 µM | >10 | <30% inhibition at 1 µM | >10000 |
| **5** | <30% inhibition at 1 µM | >10 | <30% inhibition at 1 µM | >10000 |
| **6** | <30% inhibition at 1 µM | >10 | <30% inhibition at 1 µM | >10000 |
| **7** |  |  | <30% inhibition at 1 µM | >10000 |
| **8** | <30% inhibition at 1 µM | 5.8 | <30% inhibition at 1 µM | 5800 |
| **9** | <30% inhibition at 1 µM | 5.1 | <30% inhibition at 1 µM | 5100 |
| **10** |  |  | <30% inhibition at 1 µM | >10000 |
| **11** |  |  | <30% inhibition at 1 µM | >10000 |
| **12** | 0.55 | 0.51 | 550 | 510 |
| **13** | 0.68 | 0.20 | 150 | 200 |
| **14** | <30% inhibition at 1 µM | 9.1 | <30% inhibition at 1 µM | 9100 |
| **15** | 0.91 | 6.5 | 910 | 6500 |
| **16** | 1.6 | 0.81 | 1600 | 810 |
| **17** | 1.1 | 0.91 | 1100 | 900 |
| **18** |  |  | 110 | 120 |
| **19** | 0.015 | 0.289 | 43 | 57 |
| **20** | - | - | 140 | 110 |
| **21** | - | - | 95 | 49 |
| **22** | - | - | 1500 | 3200 |
| **23** | - | - | 3400 | 5200 |
| **24** | - | - | <30% inhibition at 1 µM | >10000 |
| **25** | - | - | <30% inhibition at 1 µM | >10000 |
| **26** | - | - | 235 | 110 |
| **27** | - | - | 9 | 6 |
| **28** | - | - | 345 | 173 |
| **29** | - | - | <30% inhibition at 1 µM | >10000 |
| **30** | - | - | 57 | 110 |
| **31** | - | - | 120 | 45 |
| **32** | - | - | 52 | 65 |
| **33** | - | - | 51 | 45 |
| **34** | - | - | 8 | 11 |
| **35** | - | - | 25 | 20 |
| **36** | - | - | 1500 | 5100 |
| **37** | - | - | 880 | 780 |
