# Supplementary material for: G9a an Epigenetic Therapeutic Strategy for Neurodegenerative Conditions: From Target Discovery to Clinical Trials
Source: Med Res Rev. 2025 Jan 6;45(3):985–1015. doi: 10.1002/med.22096 (PMC11976383; doi:10.1002/med.22096)
Supplement: Supplementary file 8 — Supporting information. [file MED-45-985-s002.docx]

**Supplementary Table 2**. Biological activity of quinazoline derivatives.

| **Compounds** | **G9a IC_50_ (µM)** | **Cellular IC_50_ (µM) (ICW IC_50_)** | **Cell Toxicity EC_50_ (µM) (MTT EC_50_)** | **Tox/function Ratio (MTT EC_50_ / ICW IC_50_)** | **ALogP** | **G9a IC_50_ (nM) Radioactive assay** | **SETD8 IC_50_ (µM)** | **IC_50_ (µM)**  **SPA Assay** |
| --- | --- | --- | --- | --- | --- | --- | --- | --- |
| **38** | 0.010 | 0.52 | 1.4 | 3 | 3.3 | - |  |  |
| **39** | 0.005 | 0.47 | 1.7 | 4 | 3.2 | - |  |  |
| **40** | 0.002 | 0.34 | 4.5 | 13 | 3.5 | - |  |  |
| **41** | 0.002 | 0.10 | 0.73 | 7 | 4.7 | - |  |  |
| **42** | 0.007 | 0.093 | 0.76 | 8 | 5.0 | - |  |  |
| **43** | 0.010 | 0.22 | 2.1 | 10 | 4.5 | - |  |  |
| **44** | 0.009 | 0.058 | 0.6 | 10 | 4.4 | - |  |  |
| **45** | 0.020 | 0.10 | 5.7 | 57 | 4.2 | <2.5 |  |  |
| **46** | 0.016 | 0.15 | 7.0 | 47 | 3.7 | <2.5 |  |  |
| **47** | 0.012 | 0.081 | 11 | 140 | 4.8 | <2.5 |  |  |
| **48** | 0.11 | 0.26 | 7.6 | - | 5.3 | - |  |  |
| **49** | 0.021 | 0.18 | 13 | - | 4.2 | - |  |  |
| **50** | 0.006 | 0.026 | 3.3 | - | 5.1 | - |  |  |
| **51** | 0.004 | 0.025 | 2.8 | - | 5.5 | - |  |  |
| **52** | 0.008 | 0.054 | 4.1 | - | 4.0 | - |  |  |
| **53** | 0.011 | 0.056 | 2.9 | - | 4.4 | - |  |  |
| **54** | 0.031 | 0.24 | 6.6 | - | 4.6 | - |  |  |
| **55** | - | - | - | - | - | <2.5 |  |  |
| **56** | - | - | - | - | - | <2.5 |  |  |
| **57** | - | - | - | - | - | 26 |  |  |
| **58** | - | - | - | - | - | 14 |  |  |
| **59** | - | - | - | - | - | 9 |  |  |
| **60** | - | - | - | - | - | 3 |  |  |
| **61** | - | - | - | - | - | 4 |  |  |
| **62** | - | - | - | - | - | >26000 |  |  |
| **63** | - | - | - | - | - | >50000 |  |  |
| **65** |  |  |  |  |  |  | 7.3 |  |
| **66** |  |  |  |  |  |  | 67 |  |
| **67** |  |  |  |  |  |  | 36 |  |
| **68** |  |  |  |  |  |  | 43 |  |
| **69** |  |  |  |  |  |  | 7.9 |  |
| **70** |  |  |  |  |  |  | 7.9 |  |
| **71** |  |  |  |  |  |  | 21 |  |
| **72** |  |  |  |  |  |  | 35 |  |
| **73** |  |  |  |  |  |  | 32 |  |
| **74** |  |  |  |  |  |  | 29 |  |
| **75** |  |  |  |  |  |  | 25 |  |
| **76** |  |  |  |  |  |  | 34 |  |
| **77** |  |  |  |  |  |  | 58 |  |
| **78** |  |  |  |  |  |  | >250 |  |
| **79** |  |  |  |  |  |  | 94 |  |
| **80** |  |  |  |  |  |  | >250 |  |
| **81** |  |  |  |  |  |  | 9.2 |  |
| **82** |  |  |  |  |  |  | >250 |  |
| **83** |  |  |  |  |  |  | >250 |  |
| **84** |  |  |  |  |  |  | >250 |  |
| **85** |  |  |  |  |  |  | 37 |  |
| **86** |  |  |  |  |  |  | 37 |  |
| **87** |  |  |  |  |  |  | >250 |  |
| **88** |  |  |  |  |  |  | >250 |  |
| **89** |  |  |  |  |  |  |  | **0.067** |
| **90** |  |  |  |  |  |  |  | **0.101** |
| **91** |  |  |  |  |  |  |  | **0.472** |
| **92** |  |  |  |  |  |  |  | **3.190** |
| **113** |  |  |  |  |  |  |  | 0.013 |
| **114** |  |  |  |  |  |  |  | 0.031 |
