# Supplementary material for: G9a an Epigenetic Therapeutic Strategy for Neurodegenerative Conditions: From Target Discovery to Clinical Trials
Source: Med Res Rev. 2025 Jan 6;45(3):985–1015. doi: 10.1002/med.22096 (PMC11976383; doi:10.1002/med.22096)
Supplement: Supplementary file 9 — Supporting information. [file MED-45-985-s004.docx]

**Supplementary Table 3**. Evaluation of different substituents of quinazoline on biological activity of G9a and GLP.

|  | | | | | | |
| --- | --- | --- | --- | --- | --- | --- |
| **Compounds** | **R1** | **R2** | **R3** | **R4** | **IC_50_ (nM)** | |
|  |  |  |  |  | **GLP** | **G9a** |
| **115** |  |  |  |  | 11 | 191 |
| **116** |  |  |  |  | 18 | 465 |
| **117** |  |  |  |  | 5 | 295 |
| **118** |  |  |  |  | 17 | 356 |
| **119** |  |  |  |  | 4 | 259 |
| **120** |  |  |  |  | 6 | 177 |
| **121** |  |  |  |  | 18 | 318 |
| **122** |  |  |  |  | 30 | 550 |
| **123** |  |  |  |  | 159 | 1160 |
| **124** |  |  |  |  | 43 | 724 |
| **125** |  |  |  |  | 28 | 359 |
| **126** |  |  |  |  | 50 | 726 |
| **127** |  |  |  |  | 20 | 532 |
| **128** |  |  |  |  | 46 | 534 |
| **129** |  |  |  |  | 20 | 282 |
| **130** |  |  |  |  | 58 | 1630 |
| **131** |  |  |  |  | 24 | 151 |
| **132** |  |  |  |  | 73 | 484 |
| **133** |  |  |  |  | 23 | 343 |
| **134** |  |  |  |  | 20 | 481 |
| **135** |  |  |  |  | 23 | 508 |
| **136** |  |  |  |  | 36 | 443 |
| **137** |  |  |  |  | 77 | 956 |
| **138** |  |  |  |  | 24 | 585 |
| **139** |  |  |  |  | 135 | 1582 |
| **140** |  |  |  |  | 27 | 315 |
| **141** |  |  |  |  | 59 | 788 |
| **142** |  |  |  |  | 35 | 436 |
| **143** |  |  |  |  | 63 | 341 |
| **144** |  |  |  |  | 44 | 756 |
| **145** |  |  |  |  | 12 | 313 |
| **146** |  |  |  |  | 16 | 190 |
| **147** |  |  |  |  | 12 | 31 |
| **148** |  |  |  |  | 97 | 1060 |
| **149** |  |  |  |  | 4 | 9 |
| **150** |  |  |  |  | 341 | 1910 |
| **151** |  |  |  |  | 34 | 438 |
| **152** |  |  |  |  | 7 | 992 |
| **153** |  |  |  |  | 35 | 407 |
| **154** |  |  |  |  | 28 | 306 |
| **155** |  |  |  |  | 18 | 311 |
| **156** |  |  |  |  | 13 | 440 |
| **157** |  |  |  |  | 26 | 323 |
| **158** |  |  |  |  | 11 | 224 |
| **159** |  |  |  |  | 15 | 262 |
| **160** |  |  |  |  | 12 | 313 |
| **161** |  |  |  |  | >5000 | >6000 |
| **162** |  |  |  |  | >6000 | >6000 |
| **163** |  |  |  |  | 1230 | 3690 |
| **164** |  |  |  |  | 808 | 4070 |
| **165** |  |  |  |  | >1000 | >5000 |
| **166** |  |  |  |  | >5000 | >5000 |
| **167** |  |  |  |  | >5000 | >5000 |
| **168** |  |  |  |  | 29 | 1150 |
