# Supplementary material for: G9a an Epigenetic Therapeutic Strategy for Neurodegenerative Conditions: From Target Discovery to Clinical Trials
Source: Med Res Rev. 2025 Jan 6;45(3):985–1015. doi: 10.1002/med.22096 (PMC11976383; doi:10.1002/med.22096)
Supplement: Supplementary file 10 — Supporting information. [file MED-45-985-s008.docx]

**Table 4.** Different derivatives of quinazoline along with their activity towards GLP and HDAC1/6 target.

|  | | | | | |
| --- | --- | --- | --- | --- | --- |
| **Compounds** |  |  | **IC_50_ (nM)** | | |
|  | **R1** | **R2** | **HDAC1** | **HDAC6** | **GLP** |
| **172** |  |  | 1.3 | 51 | 11 |
| **173** |  |  | 5.8 | 16 | 21 |
| **174** |  |  | 8.4 | 131 | 97 |
| **175** |  |  | 4.2 | 12 | 11 |
| **176** |  |  | 51 | 24 | 1.6 |
| **177** |  |  | 3.2 | 17 | 9.2 |
| **178** |  |  | 89 | 13 | 1.3 |
| **179** |  |  | 94 | 50 | 190 |
| **SAHA** |  |  | 16 | 13 |  |
| **UNC0642** |  |  |  |  | 2.8 |
